# The Implementation and Evaluation of the South African Adaptation of the JOBS Program

**DOI:** 10.3389/fpsyg.2020.01418

**Published:** 2020-07-14

**Authors:** Rachele Paver, Hans De Witte, Sebastiaan Rothmann, Anja Van den Broeck, Roland Willem Bart Blonk

**Affiliations:** ^1^Optentia Research Focus Area, North-West University Vaal Triangle Campus, Vanderbijlpark, South Africa; ^2^Research Group Work, Organizational and Personnel Psychology, KU Leuven, Leuven, Belgium; ^3^Department of Work and Organization Studies, KU Leuven, Brussels, Belgium; ^4^Healthy Living, Netherlands Organisation for Applied Scientific Research, Leiden, Netherlands; ^5^Department of Human Resource Studies, Tilburg University, Tilburg, Netherlands

**Keywords:** job-search self-efficacy, amotivation, self-esteem, job-search intervention, JOBS program, Qhubekela Phambili career-enhancement program, unemployed, South Africa

## Abstract

No validated intervention that specifically addresses the psychosocial needs of unemployed people exists in the South African context. This study intends to evaluate an evidence-based job-search program, called the JOBS intervention, that is aimed at the self-efficacy, amotivation, and self-esteem related to participants searching for jobs. A quasi-experimental research design was used. Convenient samples were taken of unemployed individuals from two low-income communities (*N* = 130; experimental group = 69; control group = 61). The Qhubekela Phambili program, which is based on the JOBS program, was adapted for the South African context and was implemented over six 4-h sessions. Data collection took place pre- and post-intervention. One-way repeated measures multivariate analysis of variance was used to analyze the data. Those who had undergone the intervention showed statistically significantly higher levels of job-search self-efficacy and self-esteem. This study makes a novel contribution to the literature on the JOBS program, particularly regarding developing countries. This study showed that in a context characterized by poverty and a lack of support for the unemployed, the program also delivered promising results. It confirms previous findings that the JOBS program is suitable in a variety of labor market and economic conditions.

## Introduction

Unemployment has various adverse consequences for people, such as decreased self-esteem, a loss of personal control, and patterns of reduced (or no) job-search efforts, when unemployed for prolonged periods (Schaufeli and VanYperen, 1993; Kanfer et al., 2001; Paul and Moser, 2009; Preuss and Hennecke, 2018; Wanberg et al., 2020). Considering the undesirable effects of unemployment, the limited solutions sought from a psychosocial perspective is concerning, particularly so within the South African context (Cobb-Clark, 2015).

Labor market interventions generally focus on job creation, subsidized employment opportunities, and providing the unemployed with resources and services, such as vocational training and employment services (Kluve et al., 2017). While these programs undoubtedly assist in removing some constraints to obtain employment, a somewhat underemphasized approach to solving the employment challenge may be resource-based interventions (Malmberg-Heimonen et al., 2019) driven from a psychosocial perspective, that support the emotional and psychological needs of the participants (Price and Vinokur, 2014; Van den Hof, 2015; Paver et al., 2019a). In a meta-analysis evaluating job-search interventions, Liu et al. (2014) found that programs aimed at strengthening participants’ self-efficacy, encouraging proactive and goal-orientated behavior, teaching job search skills, and providing them with social support were more effective than interventions that did not include such components. Also, interventions that combined skills development with a motivational component were most successful in facilitating job-search success (Liu et al., 2014; Wanberg et al., 2020).

Previously conducted research agrees on the fact that unemployment should be seen within a social, more humane context and not solely as an economic burden (Donaldson and Weiss, 1998). The latent deprivation model (Jahoda, 1982) suggests that a job fulfills both manifest (remuneration) and latent functions (time structure, social contact, common goals, status or identity, and enforced activity). Likewise, Donaldson and Weiss (1998) state that employment increases people’s self-esteem and provides them with a sense of identity. Consequently, when people lose their jobs, they experience not only a loss of income but also a loss of these essential latent functions, which may negatively impact one’s psychological well-being (Jahoda, 1982). Therefore, based on these views of the importance of work, it should be considered that economic and psychological deprivation are so closely intertwined that one aspect cannot be affected without impacting the other (Møller, 1991). By applying labor market programs that solely focus on easing financial hardship (which is the status quo in the African context), psychological aspects, which may contribute to making unemployment (un)bearable, are deliberately left out of the equation.

Even though South Africa has one of the highest unemployment rates in the world (World Bank, 2018) programs implemented to assist the unemployed lack the psychosocial aspects required to have a sustainable impact (Paver et al., 2019a). An intervention designed specifically for this purpose is the JOBS program (Caplan et al., 1989; City of Johannesburg, 2018). This empirically robust preventive intervention has obtained positive results in various studies (Caplan et al., 1989; Vinokur et al., 1995; Malmberg-Heimonen et al., 2019). The purpose of this study is to implement the JOBS program, with the aim of studying whether results, similar to previous versions of the program, can be achieved, within the South African context.

## The Jobs Program

In general, the aim of JOBS program is to prevent deterioration of mental health and promotion of (re)employment (Vinokur and Van Ryn, 1993). These instilled outcomes, however, operate as a vehicle to build participants’ competence and confidence, making the real underlying goal of the program to empower them by focusing on their emotional and motivational needs and helping them to cope with their circumstances (Curran et al., 1999). This is accomplished through the trainers’ abilities to facilitate the process in which the participants acquire the intended skills while applying the underlying principles of the program. The JOBS program comprises the following underlying five principles:

*Guiding behavior.* Facilitators have the responsibility to demonstrate effective and positive behavior to participants. They provide participants with specific positive feedback and steer them away from any ineffective or negative actions or feedback.

*Social support.* Fragile participants are put at ease by creating a supportive environment that encourages unconditional acceptance by facilitators and other participants (Michigan Prevention Research Center, 2013). This entails building trust and reducing the social distance by treating participants with respect and offering positive feedback. Generating such a safe environment creates an opportunity to destigmatise unemployment and remove other associated negative connotations (Vinokur and Price, 2015). Trainers model and reinforce supportive behaviors for participants. Group exercises are designed in such a way that they provide opportunities for participants to be supportive of one another.

*Trainer referent power.* The influence trainers have on participants is based on the participants’ experience of trainers as reliable self-esteem enhancers and as referent persons whom they admire. This requires trainers to build trust and reduce social distance by providing participants with unconditional positive regard, specific positive feedback, moderate self-disclosure, and encouragement of participant self-disclosure. Facilitators also create opportunities in which they help participants to realize that their accomplishments are entirely a result of their hard work and talents (Curran et al., 1999).

*Inoculation against setbacks.* Inoculation training is a coping and group problem-solving process. It involves specific problem identification and analysis, generation of possible behavioral or cognitive responses, evaluation of responses, skill acquisition, and behavioral rehearsal, and re-evaluation. The program emphasizes an extension of this process, where the group anticipates possible barriers and, once challenges and obstacles have been identified, problem-solving skills are required to generate and evaluate potential solutions to overcome these setbacks (Curran et al., 1999).

*Active teaching/learning methods.* The program relies strongly on active learning techniques (e.g., brainstorming sessions, group discussions, problem-solving, and role-playing exercises), as opposed to didactic passive methods (Caplan et al., 1997). Facilitators refrain from providing solutions and rather foster an environment where the participants are encouraged to apply their knowledge and experience as part of the learning process (Caplan et al., 1997). The learning process is elicited through group discussions, brainstorming, and other activities. Participants spend much of their time rehearsing new skills and providing support to one another. This process seeks to maximize the effectiveness of the learning environment and to promote self-esteem and self-efficacy.

The principles mentioned above are the essence of the JOBS program. Because participants actively participate and rely on their knowledge and experience throughout the program, they tend to take ownership of their situation. Likewise, by asking the participants to suggest solutions, they are actively involved in the problem-solving process. The more it is repeated, the more natural the process becomes. The unconditional positive regard and support provided by the facilitators and the other participants also contribute to a space where participants can share experiences for others to relate to (Curran et al., 1999). Finally, the facilitators play a crucial role in the example they portray to participants.

## The Current Study

The earliest version of the JOBS program is referred to as the JOBS I intervention. Findings from the original JOBS intervention demonstrated that some components of the program contributed more prominently to the positive outcomes achieved (Vinokur et al., 1995). This led to the development of the JOBS II intervention. One of the components that inspired the changes made in the revised program was a stronger focus on participants’ sense of mastery – comprising job-search self-efficacy, locus of control, and self-esteem (Vinokur et al., 2000). Because a sense of mastery played such a prominent role in the JOBS intervention, the current paper aims to determine the effectiveness and the role of the various components, job-search self-efficacy, locus of control, and self-esteem.

Unemployment is often accompanied by humiliation and feelings of inferiority and incompetence (Curran et al., 1999). Studies showed that providing jobseekers with coping resources, such as the confidence and ability to effectively seek for employment, overcoming feelings of helplessness, and building their self-esteem can be vital in their search for employment (Van Ryn and Vinokur, 1992; Vinokur et al., 1995, 2000; Price and Vinokur, 2014). Successfully obtaining a new job largely depends on an individual’s ability to apply job-search strategies effectively (Price et al., 2002a) hence the emphasis placed on job-search self-efficacy within the JOBS program.

Bandura describes self-efficacy as a person’s belief that they can be successful (Bandura, 1986). Because self-efficacy is context-specific, job-search self-efficacy refers to individuals’ confidence in their ability to perform job-seeking activities (Vinokur and Caplan, 1987; Saks and Ashforth, 1999). The JOBS program likely enhances the perceptions of job search self-efficacy because it teaches new skills, and it tries to embed the newly taught behaviors into people’s skill set through consistent positive reinforcement from the facilitators. The acquisition of these resources is accomplished through the empowerment of participants to confidently and persistently engage in job-seeking undertakings (Vinokur et al., 2000). The JOBS intervention is specifically designed to maximize the job-search skills of the job seekers and to enhance and maintain their motivation (Price and Vinokur, 2014) building their confidence to seek employment. Therefore, the following hypothesis is formulated:

*Hypothesis 1.* After taking part in the JOBS program, participants from the experimental group will report higher levels of job-search self-efficacy, compared to their control group counterparts.

The contexts in which the JOBS program has been implemented consist mainly of Western countries and differ quite significantly from the South African context. One significant difference may be the prolonged periods of unemployment experienced by job seekers. Unemployment, but perhaps even more so, long-term unemployment, may contribute to people feeling discouraged about re-entering the labor market (Van der Vaart et al., 2018). This discouragement intensifies feelings of helplessness and loss of personal control (Curran et al., 1999). A loss of control (or perceptions that chance is a controlling factor in finding employment) among unemployed is associated with longer periods of unemployment (Houssemand et al., 2019). While, a loss of personal control may be common among unemployed persons, perhaps more uncommon is the fact that 32% of the unemployed in South Africa have been identified as being amotivated (Van der Vaart et al., 2019) implying that they are neither intrinsically nor extrinsically motivated and may experience the relative absence of motivation (Rotter, 1966; Ryan and Deci, 2000). A fundamental component of the JOBS program is inoculating participants against setbacks. This entails that participants anticipate potential challenges or difficulties, generate possible solutions to overcome these obstacles or barriers, and acquire the necessary skills to deal with the setbacks (Hobfoll, 1989; Vinokur and Schul, 1997). What is essential is that these are the participants’ *own* solutions. Thus, they are cultivating control that empowers them to take ownership of their situation (Deci and Ryan, 1985; Curran et al., 1999). It seems reasonable to ascertain that these unemployed individuals, who are particularly prone to be amotivated, may benefit from an intervention such as the JOBS program. Therefore, the second hypothesis is as follows:

*Hypothesis 2.* After taking part in the JOBS program, participants from the experimental group will report lower levels of amotivation, compared to their control group counterparts.

Again, drawing on Jahoda’s work, a loss of self-esteem is one of the deprivations experienced when losing one’s job (Jahoda, 1982). To successfully find employment, self-esteem is considered essential to remaining motivated and to persevere in job search behavior (Caplan et al., 1989). Rosenberg describes self-esteem as the extent to which one’s attitude is favorable or unfavorable toward him- or herself (Rosenberg, 1965). It is understandable that such a perception may be influenced by one’s employment status, perhaps even more so for individuals who have never held a job before. The JOBS intervention protocol relies heavily on confidence-boosting techniques and empowering participants by giving them opportunities to become aware of, display, and build their personal resources (Price et al., 2002b). Activities were designed to have participants experience success through trainers providing participants with sincere and specific praising comments on the behavior. The purpose of the support provided by the facilitators and other participants is to establish a sense of belongingness and to enhance participants’ self-esteem (Caplan et al., 1989). Finally, the third hypothesis derives as follows:

*Hypothesis 3.* After taking part in the JOBS program, participants from the experimental group will report higher levels of self-esteem, compared to their control group counterparts.

## Materials and Methods

### Research Design

**FIGURE 1:**
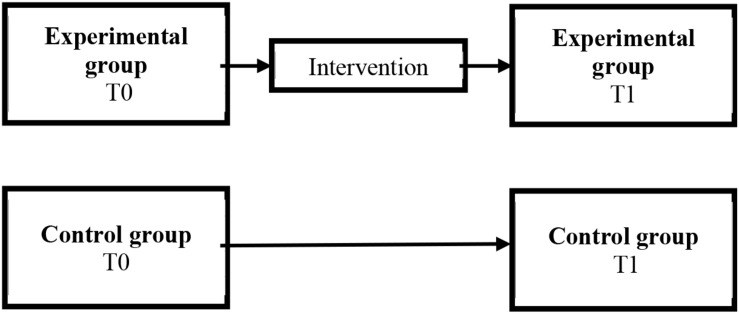
The quasi-experimental research design.

To evaluate the impact of the JOBS intervention, a quasi-experimental research design was used (see Figure 1). Participants were randomly assigned to an experimental or control group. Both groups were tested at two intervals (Time 0 and Time 1), with the experimental group undergoing the intervention between the two measurements. The outcomes of the experimental group were compared with the outcomes of the control group, to determine the impact of the intervention.

### Intervention

#### Qhubekela Phambili Career-Enhancement Program

To better understand the mechanisms of the JOBS program and its effects, Paver et al. (2019b) conducted a literature review of the JOBS program and variations thereof. The current study is executed based on suggestions made to effectively implement and evaluate the JOBS program (see Supplementary Material; Paver et al., 2018). The name of the South African version of the JOBS program is Qhubekela Phambili, which means “moving forward” in IsiZulu.

#### Content

Striking a balance between maintaining fidelity to evidence-based practices and adapting the program to suit local circumstances can be problematic (Price and Vinokur, 2014). Although some content-related changes were made, none of the changes influenced the theoretical foundation of the program. Due to slow economic growth and limited job opportunities in the labor market, paid employment is not always a feasible option (Price and Vinokur, 1995). Also, the lack of means to travel from their communities to a job that unemployed people experience makes it challenging. Therefore, a critical element that was incorporated into the Qhubekela Phambili program was a self-employment/entrepreneurial component. The main content-related difference is that the original JOBS program aims to link participants’ strengths, passions, values, network, action plans, and job-search strategies to a job within the formal sector. Participants are encouraged to develop action plans to which they commit themselves, such as developing a strong network of people and identifying job leads. The Qhubekela Phambili program draws participants’ attention to the needs and opportunities within their community and to accordingly align their identified strengths, passions, values, network, and opportunity-seeking strategies to create and utilize opportunities. Their action plan and what they commit to is, therefore, not limited to seeking job leads and opportunities within the formal sector, but is also expanded to identify and commit to self-employment opportunities. An outline of the program content is presented in Table 1.

**TABLE 1:** An outline of the Qhubekela Phambili career-enhancement program.

Topics	Duration
**Session 1: Who am I, and what am I good at?**	
Introduction (includes time for questionnaire)	140 min
What do I stand for?	50 min
What am I good at?	40 min
Conclusion	15 min
**Session 2: Exploring my passions, my values, and my future**	
Welcome	15 min
What are my passions?	30 min
What are my values?	75 min
Distinguishing between employment and entrepreneurship	40 min
Linking my attributes to my career	35 min
Conclusion	15 min
**Session 3: Exploring opportunities and finding ways to realize them**	15 min
Welcome	45 min
Identifying possibilities applicable to me	50 min
My future	90 min
Developing my career	15 min
Conclusion	
**Session 4: What resources do I have?**	15 min
Welcome	125 min
My network plan	30 min
Resources in my community	15 min
Conclusion	
**Session 5: Resources within myself**	15 min
Welcome	40 min
Communication skills and professional conduct	50 min
My goals	20 min
What is holding me back?	105 min
Conclusion (includes time for questionnaire)	15 min
Closing ceremony	
**Session 6: Return day**	35 min
Welcome and looking back	60 min
My goals/commitments	105 min
Conclusion (includes time for questionnaire)	

#### Training of Facilitators

The first author who led and coordinated the project was trained in the JOBS program in the Netherlands. This was done to ensure that the program will be delivered at an acceptable standard and to ensure the reliability and validity of the JOBS intervention. Additionally, an international trainer with vast experience in conducting the JOBS program was invited to present a train-the-trainer workshop to selected facilitators in South Africa.

Six facilitators were trained in the program. These candidates were selected on the premise that they have a background in psychology, social work, industrial psychology, or a related field. Facilitators also had to be experienced in working with people in similar conditions, possess good facilitation skills, such as speaking and listening skills, giving feedback, facilitating, understand group processes and dynamics, and able to deal with conflict constructively (Price and Vinokur, 1995). The train-the-trainer program was conducted over five 8-h sessions. Practical exercises were integrated into these sessions so that where trainees had the opportunity to practice the relevant skills. These sessions aimed to ensure that the facilitators were comfortable with the content and the underlying processes of the program. During the Qhubekela Phambili program, facilitators met daily to share their experiences and to deal with topics and possible issues that may have surfaced during the sessions (Price and Vinokur, 1995).

### Participants and Data Collection Procedures

Unemployed individuals from townships in two geographical areas in Gauteng, namely Orange Farm and Boipatong, were approached to participate in the study. Several inclusion criteria were used to select participants. Participants had to be unemployed South African citizens, aged between 18 and 55. They had to reside in either Boipatong or Orange Farm. They also had to show an adequate understanding of English. Participants from the same regions, who previously participated in an unemployment research conducte by Du Toit et al. (2018) and Van der Vaart et al. (2018) were contacted to participate. From the 867 participants previously involved one of the studies (Van der Vaart et al., 2019) only 354 met our inclusion criteria. An additional 60 people, who were previously involved in the study conducted by Du Toit et al. (2018) were approached to participate. The identified people were contacted, via telephone. During each call the purpose of the program, as well as basic information such as the dates, time, duration, and location of the program. Based on the potential participants’ adherence to the inclusion criteria and their willingness to participate they were divided into two groups randomly – an experimental and a control group.

Initially, 414 people, selected from previous studies, were invited to participate in the workshop. However, among both groups some participants who had agreed to participate failed to show up. Attrition of participants in social interventions is also unavoidable (Vinokur et al., 1991a). In some cases, the percentage of “no shows” was up to 75% of the group. In these cases, community leaders who work with the unemployed were asked for assistance to recruit additional participants. Although the inclusion criteria were communicated to leaders, non-compliant participants were still invited. The requirement the majority did not meet was the completion of Grade 12. It did not seem to have any impact on the process. There were two cases where participants were not able speak to English fluently. Although facilitators could communicate with them in other languages, completing the questionnaires seemed to be a barrier. They were therefore omitted from the study.

The Qhubekela Phambili program consisted of five 4-h sessions in 1 week with a return day 4 weeks after the program. Two experimental groups attended the workshop per week (morning and afternoon group). At the same time, two control groups were asked to complete the research questionnaire. The intervention program was executed over 2 weeks and included four experimental groups and four control groups. Both groups (intervention and control) were asked to complete a pre-test survey on the first day of the program and a post-test survey on the last day of the program (although the control group did not receive the intervention). The attendance and dropout of participants are reported in Table 2.

**TABLE 2:** Attendance and dropout of participants (*N* = 130).

	Morning session	Afternoon session

	(08:00–12:00)	(13:00–17:00)
Week 1	*Experimental group 1 (attended the program)*	*Experimental group 2 (attended the program)*
	Invited to participate *n* = 25	Invited to participate *n* = 25
	Arrived for program *n* = 19	Arrived for program *n* = 5
	Final number participated *n* = 19	Additional participants recruited *n* = 19
		Final number participated *n* = 14
	*Control group 1 (completed the survey)*	*Control group 2 (completed the survey)*
	Invited to pre-test *n* = 25	Invited to pre-test *n* = 25
	Arrived for pre-test *n* = 25	Arrived for pre-test *n* = 16
Week 2	*Experimental group 3 (attended the program)*	*Experimental group 4 (attended the program)*
	Invited to participate *n* = 20	Invited to participate *n* = 20
	Arrived for program *n* = 19	Arrived for program *n* = 3
	Final number participated *n* = 19	Additional participants recruited *n* = 15
		Final number participated *n* = 18
	*Control group 3 (completed the survey)*	*Control group 4 (completed the survey)*
	Invited to pre-test *n* = 20	Invited to pre-test *n* = 20
	Arrived for pre-test *n* = 18	Arrived for pre-test *n* = 17

The characteristics of the participants are displayed in Table 3.

**TABLE 3:** Characteristics of the experimental (*N* = 69) and control groups (*N* = 61).

	Experimental group		Control grouph	
Characteristics	Total	%	Total	%
**Gender**				
Male	27	39%	23	40%
Female	42	61%	35	60%
**Age**				
Below 20	1	1%	3	7%
20–29	36	52%	27	47%
30–39	23	33%	16	28%
40–49	6	9%	9	22%
50–59	2	3%	3	5%
**Race**				
Black	69	100%	41	100%
White	0	0%	0	0%
Indian	0	0%	0	0%
Colored	0	0%	0	0%
**Education level**				
Less than Grade 12	21	30%	23	40%
Grade 12	37	54%	25	43%
National Certificate	10	14%	4	7%
National Diploma/Degree	1	1%	6	10%
**Duration of unemployment**				
Less than 3 months	9	13%	3	7%
3–5 months	5	7%	7	12%
6–11 months	5	7%	4	6%
1–2 years	8	12%	16	28%
2–5 years	27	39%	12	21%
More than 5 years	15	22%	16	28%

**Data was collected using self-administered questionnaires on two occasions. Questionnaires were completed with pen and paper and took ∼40 min. In cases where participants had difficulty understanding the survey questions, the facilitators explained them, without changing the meaning of the questions. A participation number was allocated to participants to identify them in the subsequent data collection rounds. Therefore, participation was not completely anonymous, but questionnaires did not contain any confidential information. Furthermore, facilitators were asked to take note of their experiences and the observed behaviors of the participants after each session. All notes and observations were used as qualitative information to be referred to in the explanation of the findings.**

### Measuring Instruments

#### Biographical Information

A questionnaire to determine the biographical characteristics of the participants was used. Characteristics such as gender, age, race, level of education, and duration of unemployment were asked in this questionnaire.

#### Job-Search Self-Efficacy

The job-search self-efficacy measure was developed in earlier investigations of the JOBS program (Vinokur et al., 1991b; Vinokur and Schul, 2002; Lefcourt, 2014). It consists of six items. Participants were asked how confident they felt executing tasks related to finding a job (i.e., “How confident do you feel about completing a good job application and CV?”). Items were rated on a five-point frequency scale ranging from not at all confident (l) to a very confident (5). The Cronbach’s alpha coefficient for this index was α = 0.79 (Time 0) and α = 0.74 (Time 1).

#### Amotivation

The self-regulation questionnaire job-searching scale (Vansteenkiste et al., 2004) was used to measure amotivation (Vansteenkiste et al., 2005). This scale consists of 10 items and intends to measure individuals’ motives not to search for employment (i.e., “I do not look for a job because I am tired of looking for a job”). Items were measured on a five-point Likert scale, ranging from strongly disagree (1) to strongly agree (5). The scale showed acceptable internal consistency (α = 0.82, Time 0; 0.85, Time 1).

#### Self-Esteem

The Rosenberg self-esteem scale (Rosenberg et al., 1995) was utilized to measure respondents’ positive and negative feelings about themselves (i.e., “On the whole, I am satisfied with myself”). This scale consisted of 10 items and was measured on a five-point Likert scale, ranging from strongly disagree (1) to strongly agree (5). After analyzing the reliability coefficient of the scales, some problematic items were identified. The loading of item 8 (“I wish I could have more respect for myself”) in the self-esteem scale was not significant and was therefore deemed not suitable for inclusion in further analyses. Cronbach’s alpha coefficient for this scale was 0.67 (Time 0), and 0.71 (Time 1).

#### Intervention Indices

Participants’ experience and perception of the program were measured to indicate the intervention’s integrity and strength. The measure consisted of six multi-item indices, rated on a five-point scale. The following aspects of the intervention were included: Trainer support was measured with five items and group support with five items. The facilitators and other group participants were rated on their warmth, expertise, and helpfulness (1 = most negative rating; 5 = most positive rating). Both active learning and job-search skills were answered on a frequency scale that varied from 1 = not at all to 5 = a great deal and were measured with five items. Example items are “During the workshop, to what extent do you feel that you could share your experiences?” (active learning) and “During the workshop, to what extent do you feel that the trainers and other group members helped you to identify possible job opportunities?” (job-search skills). An example item of inoculation against setbacks is “Do you anticipate difficulties and setbacks during your job-search?” which was answered from 1 = very few to 5 = very much and measured with three items. Learning experience ranged from 1 (improved to a very small extent) to 5 (improved to a very great extent). It was measured with seven items (i.e., “To what extent do you feel that the workshop has prepared you to conduct interviews?”). The reliability of these scales ranged from 0.66 to 0.91.

### Statistical Analysis

SPSS version 25.0 was used to analyze the data (IBM Corp, 2017). The validity and reliability of the program was established by (i) using multivariate analysis of variance (MANOVA) to report on participant randomization; and (ii) examining mean scores of the program’s indices to determine the integrity and strength of the intervention. Secondly, Cronbach alpha coefficients were computed to establish the internal consistency of the constructs (Clark and Watson, 1995). Correlation coefficients were used to determine the relationship between the measured variables, the confidence interval level was set at 95%, to indicate statistical significance. Practical significance of the correlation coefficients was set at a cut-off point of 0.30 (medium effect) and 0.50 (large effect, Cohen, 1988). A one-way repeated measures multivariate analysis of variance (MANOVA) was used to study whether the pre- (T0) and post-test (T1) means differ between the experimental and control groups. If the one-way repeated measures MANOVA were statistically significant, we analyzed which of the groups differed in terms of the combined dependent variables and which of the variables were statistically significant. Listwise deletion were used to deal with missing data.

### Ethical Considerations

Ethical clearance was obtained from the Humanities and Health Research Ethics Committee (HHREC; NWU-HS-2018-0006). Based on these ethical considerations, much attention and effort were paid to ensure a program that is as accessible and resourceful as possible for the participants. The program was presented at a venue at the university. Participants were transported by bus from townships to the university and back. The Flemish Interuniversity Board, the funding agency for the project, paid the costs of the transport. The venue provided for a conducive atmosphere that promotes capacity building. Furthermore, participants received food parcels on each day of the workshop, a gift voucher, and an attendance certificate. An Unemployment Research Advisory Board (URAB) was established to advise the researchers on the development and implementation of the program in the two townships. Lastly, participants from the control groups (*n* = 61) were also invited to undergo the training to ensure that all participants benefit equally. This training took place 1 week after the experimental groups had undergone their training.

## Results

### Preliminary Analyses

To ensure acceptable validity and reliability of the program, previous JOBS studies used two types of measures, namely testing the integrity of randomization, and the strength of the program (Vinokur et al., 1995). The first check to determine the validity of the program is to determine whether the statistical analyses were conducted on a randomized (true) experimental design. This is determined by comparing the demographic and other tested variables of the experimental and control conditions at baseline to identify possible differences. If no significant differences are found, the integrity of randomization can be confirmed. Multivariate analysis of variance (MANOVA) were used to assess whether the participants in the condition groups (experimental and control groups; *n* = 130), at baseline, differed from participants who randomly dropped out of the program (*n* = 17). A significant difference between the level of amotivation of the two conditions and the dropout group was found [*F*(1, 147) = 4.98; *p*< 0.01; η = 0.06]. Those in the condition groups scored lower levels of amotivation (*M* = 2.47; *SD* = 0.76), compared to those who dropped out (*M* = 3.04; *SD* = 0.78). There were no significant differences between conditions for gender [*F*(1, 144] = 0.89; *p* < 0.36; partial η^2^ = 0.01), age [*F*(1, 144) = 0.27; *p* < 0.60; partial η^2^ < 0.01], race [*F*(1, 144) = 0.89; *p* < 0.36; partial η^2^ < 0.01], education [*F*(1, 144) = 0.30; *p* < 0.58; partial η^2^ < 0.01], or duration of unemployment [*F*(1, 144) = 0.03; *p* < 0.86; partial η^2^ < 0.01]. Participants who dropped out of the program were excluded from further the analyses due to the lack of data at Time 1.

To test for random sampling between the experimental and control groups, MANOVA was conducted. Baseline scores of the condition groups’ demographic variables, job-search self-efficacy, amotivation and self-esteem were compared. Supporting the successfulness of the random assignment, there were no significant differences between conditions for gender [*F*(1, 130) = 0.11; *p* < 0.74; partial η^2^ < 0.01], age [*F* (1, 130) = 1.18; *p* < 0.28; partial η^2^ < 0.01], race [*F*(1, 130) = 2.27; *p* < 0.13; partial η^2^ = 0.02], education [*F* (1, 130) = 0.12; *p* < 0.73; partial η^2^ < 0.01], or, duration of unemployment [*F*(1, 130) = 0.89; *p* < 0.36; partial η^2^ = 0.01]. Given the absence of significant *a priori* differences between the two conditions there is no need to control for any variables at baseline (Time 0), when testing the effectiveness of the intervention.

The second check is to test the strength and integrity of the intervention. Participants were asked to evaluate their experience of facilitators and the program. These evaluations are used to determine whether various intervention elements (trainer support, group support, active learning methods, job-search skills, inoculation against setbacks, and learning experiences) had been implemented and had operated as designed (Vinokur et al., 1996). Participants experienced the program as psychologically and socially positive, based on the mean scores of the intervention indices.

According to Field (2013) there are several assumptions need to be assessed prior to the analyses of inferential statistics. Some of the most common assumptions include independent random sampling, normality, linearity, equality of variance, and homogeneity of variance-covariance. Firstly, integrity of randomization has already been confirmed. Secondly, to test if the data was normally distribution an inspection of the Q-Q plots and histograms of the post-test scores on the three dependent variables showed some deviations of normality. Therefore, the results should be interpreted with caution (Field, 2013). Since, MANOVA is sensitive to outliers, Q-Q plots were used to determine whether the sample included outliers, no was outliers were detected. Thirdly, to test the linear relationship between the independent and dependent variables correlation coefficients were used. Table 4 shows the correlations between all study variables. Some relationships are worth mentioning. Several statistically significant relationships are worth mentioning: Self-esteem Time 1 showed a positive statistically significant negative effect (with medium effect) with the condition variable; participants’ age were significantly related to their duration of unemployment; job-search self-efficacy at Time 0 is significantly related to job-search self-efficacy Time 1 (with medium effect); likewise, amotivation at Time 0 is significantly related to amotivation Time 1 (with medium effect), and lastly, self-esteem Time 1 is significantly correlated with job-search self-efficacy Time 1 (with medium effect) and amotivation Time 1 (with medium effect). No signs of multicollinearity are evident, as measured variables were moderately correlated with each other.

**TABLE 4:** Correlations matrix of biographic and dependent variables (*N* = 130).

Construct	Mean	*SD*	1	2	3	4	5	6	7	8	9	10	11
1. Condition	1.47	0.50											
2. Gender	1.60	0.49	–0.03										
3. Race	1.05	0.37	0.13	–0.15									
4. Education	1.87	0.82	0.01	–0.02	–0.06								
5. Age	31.15	8.50	0.10	–0.15	0.08	−0.23**							
6. Duration of unemployment	4.28	1.58	0.04	0.11	0.02	–0.14	0.31**						
7. Job-search self-efficacy T0	3.92	0.76	0.07	–0.13	–0.03	–0.03	0.09	−0.24**					
8. Amotivation T0	2.46	0.75	–0.07	–0.02	–0.01	–0.17	0.13	0.16	–0.09				
9. Self-esteem T0	3.91	0.60	0.04	–0.03	0.09	0.11	–0.08	–0.14	0.28**	−0.24**			
10. Job-search self-efficacy T1	4.10	0.72	−0.21*	–0.12	0.07	–0.10	–0.08	–0.16	0.47**	0.05	0.10		
11. Amotivation T1	2.41	0.80	–0.02	0.01	0.03	−0.23**	0.15	0.15	–0.13	0.44**	−0.28**	–0.07	
12. Self-esteem T1	4.02	0.61	−0.40**	0.06	–0.09	0.12	−0.22*	–0.13	0.14	0.01	0.24**	0.35**	–0.33**

**p < 0.01 statistically significant; *p < 0.05 statistically significant. r < 0.30 is practically significant (medium effect); r < 0.50 is practically significant (large effect).

Fourthly, to test whether the variances of the differences between conditions are equal repeated measures MANOVA was used. Repeated measures MANOVA requires sphericity and Mauchly’s test evaluates if this holds. The sphericity assumption was met by our data because the repeated measure variables had only two levels. Finally, homogeneity of variance-covariance was tested with Box’s M test of equality of covariance homogeneity of variance. Box’s M (13.17) was not significant (*p* > 0.05), therefore, the assumption is not violated, and Wilk’s Lambda is an appropriate test to use. The results show that there are no are significant differences among the experimental and control groups on a linear combination of the three dependent variables at baseline [Wilks’ Lambda (Λ) = 0.99, *F*(3, 122) = 0.46, *p* = 0.71, η = 0.14]. Equality of variance was assessed with utilizing Levene’s test, which uses the F-test, none of the variables’ were significant [job-search self-efficacy: *F*(1, 130) = 2.31, *p* = 0.13; amotivation *F*(1, 130) = 1.45, *p* = 0.24; self-esteem: *F*(1, 130) = 0.40, *p* = 0.85]; therefore the assumption of equality of variance is met.

### Effects of the Intervention

To examine for statistical differences between the included dependable variables (job-search self-efficacy, amotivation and self-esteem), by an independent grouping variable (experimental and control conditions), a one-way repeated measures MANOVA was conducted.

**FIGURE 2:**
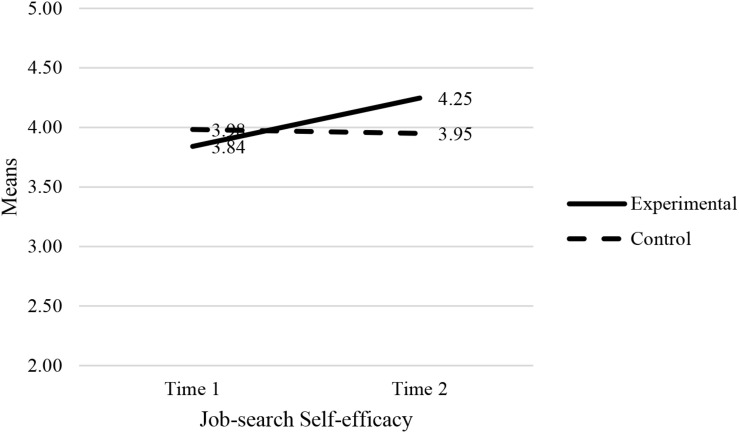
Estimated marginal means of job-search self-efficacy.

**FIGURE 3:**
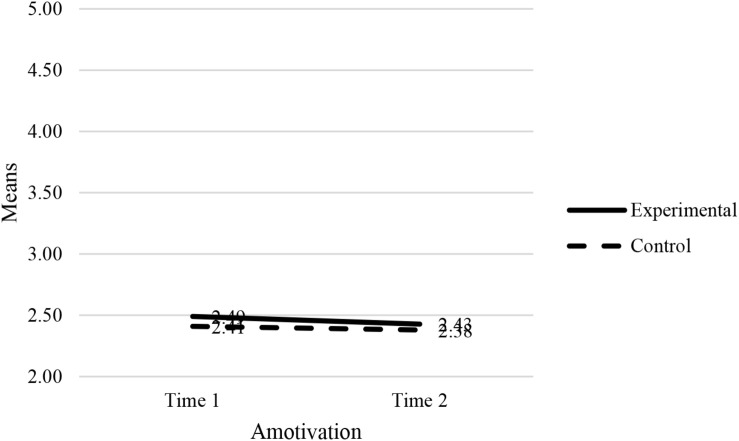
Estimated marginal means of amotivation.

**FIGURE 4:**
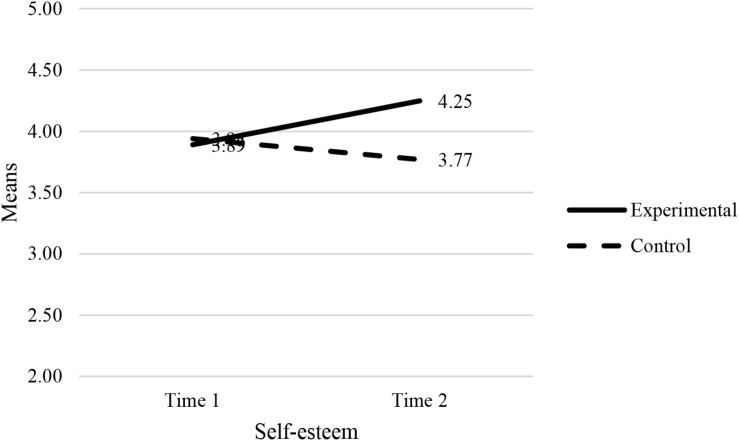
Estimated marginal means of self-esteem.

The results showed a statistically significant interaction effect between the experimental condition (experimental group vs. control group) and the combined dependent variables (job-search self-efficacy, amotivation and self-esteem): [*F*(3, 124) = 8.16, *p* < 0.001, Wilk’s Λ = 0.84, partial η^2^ = 0.17]. This was a medium effect (Cohen, 1988) where 17% of the variance is explained. Univariate analyses showed that the effects of two dependent variables (job-search self-efficacy and self-esteem) were statistically significant: job-search self-efficacy (*F* = 11.50, *p* < 0.001, partial η^2^ = 0.08) and self-esteem (*F* = 18.08, *p* < 0.001, partial η^2^ = 0.13). The F-test of amotivation was not statistically significant: *F* = 0.06, *p* >0.05. The plots in Figures 2–4 confirm that the effect for the combined dependent variable is clearly different for the experimental and control groups.

Next, we tested the effects of combined dependent variables (job-search self-efficacy, amotivation and self-esteem) for the experimental group (EG) and control group (CG) separately. The following results were obtained: *F*_*EG*_ (3, 64) = 13.56, *p* < 0.001, Wilk’s Λ = 0.61, partial η^2^ = 0.39); *F*_*CG*_ (3, 58) = 0.95, *p* > 0.5, Wilk’s Λ = 0.95).

Univariate analyses in the experimental group showed that the effects of two dependent variables (job-search self-efficacy and self-esteem) were statistically significant: job-search self-efficacy (*F* = 18.62, *p* < 0.001, partial η^2^ = 0.22) and self-esteem (*F* = 31.91, *p* < 0.001, partial η^2^ = 0.33). The F-test of amotivation was not statistically significant: *F* = 0.44, *p* > 0.05. The pairwise comparisons (of T0 and T1 scores) for the experimental and control groups are reported in Table 5. Univariate analyses in the control group showed that the effects of all three dependent variables were statistically insignificant: job-search self-efficacy (*F* = 0.08, *p* > 0.05), amotivation (*F* = 0.05, *p* > 0.05), and SE (*F* = 2.27, *p* > 0.05).

**TABLE 5:** Means, standard errors and pairwise comparisons for the experimental and control conditions.

Variable	Mean (SE)		Difference	SE	*p*	95% CI
Experimental group	T0	T1				
Job search self-efficacy	3.84 (0.07)	4.25 (0.10)	−0.41*	0.10	0.00*	[–0.66,–0.22]
Amotivation	2.49 (0.09)	2.43 (0.10)	0.06	0.09	0.51	[–0.12, 0.25]
Self-efficacy	3.89 (0.07)	4.25 (0.05)	−0.36*	0.06	0.00*	[–0.49,–0.24]

**Control group**	**T0**	**T1**				

Job search self-efficacy	3.98 (0.09)	3.95 (0.11)	0.03	0.09	0.77	[–0.15, 0.19]
Amotivation	2.41 (0.11)	2.38 (0.11)	0.03	0.12	0.83	[–0.21, 0.26]
Self-efficacy	3.94 (0.08)	3.77 (0.09)	0.17	0.11	0.14	[–0.05, 0.39]

*p < 0.001.

Therefore hypothesis 1, namely that after taking part in the JOBS program, participants from the experimental group will report higher levels of job-search self-efficacy compared to their control group counterparts, is supported.

The second hypothesis of this study was that after taking part in the JOBS program, participants from the experimental group would report lower levels of amotivation, compared to their control group counterparts. The results of the MANOVA showed that the mean amotivation score was not statistically significantly higher in the experimental group, when compared to their counterparts. As a result, hypothesis 2 is not supported.

The third hypothesis of this study was that after taking part in the JOBS program, participants from the experimental group will report higher levels of self-esteem, compared to their control group counterparts. The results of the MANOVA showed the experimental group showed statistically significantly higher mean scores for self-esteem (compared to the control group). Consequently, hypothesis 3 was supported.

## Discussion

To date, much research has been conducted on the psychological consequences of being unemployed. Yet, when it comes to practice, employability interventions seldom include crucial psychosocial aspects to assist the unemployed, particularly so in South Africa. One program addressing the gap in literature and practice is the JOBS intervention. Due to the encouraging results, several JOBS derivatives have been implemented globally (Price and Fang, 2002; Vuori et al., 2002; Barry et al., 2006; Shirom et al., 2008; Brenninkmeijer and Blonk, 2011), however, mainly in developed countries. The lack of similar interventions in South Africa, together with the successful replication of the JOBS program, created an opportunity to explore whether such an intervention will be effective in the South African context. This paper intended to apply a South African version of the JOBS program, called the Qhubekela Phambili career-enhancement program. Suggestions pertaining to the implementation and evaluation of the JOBS program made in Paver et al. (2019b) were adhered to.

Preliminary analyses were conducted to determine whether the Qhubekela Phambili program was valid and reliable. Two aspects, randomization of intervention conditions and strength and integrity of the program, were considered. Given that no significant differences were found between the experimental and control group at baseline, the integrity of randomization of the experimental and control groups was fully preserved. Also, the strength and integrity of the program was fully preserved, as the Qhubekela Phambili program adhered to the research-based principles of the JOBS program. An analysis of the working elements of the JOBS program revealed that a sense of mastery, comprising job-search self-efficacy, amotivation, and self-esteem, is one of the most predictive elements of positive outcomes in the JOBS program (Vinokur and Schul, 1997; Vinokur and Price, 2015). Therefore, the general aim of the paper was to establish whether the Qhubekela Phambili intervention affected participants’ levels of job-search self-efficacy, amotivation, and self-esteem.

The first hypothesis, and also one of the most significant findings to emerge from this study, was that the Qhubekela Phambili intervention succeeded in increasing participants’ job-search self-efficacy levels. Experimental group participants scored significantly higher in job-search self-efficacy, compared to their control group counterparts. This finding is consistent with previous empirical studies reporting that the JOBS program can enhance participants’ job-search self-efficacy. Increases in job-search self-efficacy is a crucial finding, as it has previously been related to sustained job-search efforts, despite repeated disappointments (Van Ryn and Vinokur, 1992; Choi et al., 2003).

The second aim of the study was to determine whether participants would show lower levels of amotivation after the Qhubekela Phambili workshop. No significant changes in participants’ levels of amotivation were found. This finding may be somewhat concerning, as the only variable to differ significantly differ between the condition and dropout groups was amotivation. Possible explanations for this finding may be found in the literature. It has previously been noted that the adverse effects of unemployment on mental health are larger among the long-term unemployed than the short-term unemployed (McKee-Ryan et al., 2005). Bearing in mind that 75% (18% longer than a year, 31% between 2 and 5 years, and 26% longer than 5 years) of the current study’s sample have been unemployed for longer than 12 months, severe feelings of discouragement, hopelessness, and a lack of personal control may occur. A qualitative study conducted among the unemployed in South Africa testified that due to abundant unsuccessful job-search efforts, the unemployed often develop a passive attitude and a sense of learned helplessness (Du Toit et al., 2018).

Earlier literature found that amotivation is negatively related to job-search efforts due to the jobseekers’ lack of motivation to persist (Vansteenkiste et al., 2004). Considering that feelings of helplessness may be so deeply embedded, perhaps a need exists to foster an intrinsically inclined motivation among participants. While much attention is given to inoculating participants against possible setbacks, to truly change such behavior, using the same principles to emphasize the importance of taking ownership of one’s reality may deliver promising results. This is an important finding considering the fact that it was previously reported that, in a sample of unemployed individuals, 32% reported to be amotivated (Van der Vaart et al., 2018).

The third hypothesis of this study was that participants would show higher levels of self-esteem after attending the program. Indeed, the program demonstrated the capability to increase participants’ self-esteem. As hypothesized, after taking part in the JOBS program, participants from the experimental group reported higher levels of self-esteem, compared to their control group. This finding is in line with previous research that suggests that the intervention provided the participants with the intended support to raise their self-esteem (Caplan et al., 1989; Vinokur et al., 1995; Vuori and Silvonen, 2005).

### Limitations and Recommendations

Several limitations of this study need to be acknowledged. First, only self-report measures were used. While self-report measures are considered the most appropriate for assessing perceived behavioral constructs, efforts to study such constructs more objectively may yield significant findings. Second, it was not possible to investigate the measurement invariance of the measures of the three dependent variables in this study because of the small sample sizes (Cheung et al., 2011). Future research should focus on the measurement invariance of longitudinal variables.

In social interventions, non-participation is a reality (Vinokur et al., 1991a). In previous studies on the JOBS program to preserve the integrity of a randomized experimental design, dropout participants were kept in the experimental group – it is believed to allow for stronger conclusions. Yet, it is reported that such a design yields conservative estimates of the achieved effects (Caplan et al., 1989). Since follow-up data from dropout participants in the current study was not available, the integrity of randomization was not fully preserved.

Another potential weakness in this investigation may be the number of no-show (54%) and dropout (14%) participants. Those who participated in the first round of surveys and then did not return, reported significantly higher levels of amotivation, compared to participants in the experimental conditions. Though previous research has shown that the people who need the intervention the most were indeed the ones who participated (Price et al., 1992) this is perhaps not the case in the current study. It begs the question of what the program could have achieved if targeted at this possibly more vulnerable group. Therefore, a need exists to explore improved recruitment strategies in future endeavors.

To be able to compare the findings of the current study with previous studies conducted on the JOBS program, similar dependent variables (job-search self-efficacy, amotivation, and self-esteem) were measured. However, given that the program was adjusted to also focus on entrepreneurship, an additional measure or an adapted version of the job-search self-efficacy questionnaire, not limited to job-search self-efficacy, but also determining a broader self-efficacy concept may have been valuable.

According to Price et al. (1992) participants with a higher risk of depression were more likely to benefit from the intervention. Consequently, a prospective screening mechanism was used to identify potential participants. Because 69% of the unemployed have been unemployed for longer than a year (Statistics South Africa, 2018) and that ∼70% have reported to experience unemployment as feeling desperate or discouraged (Van der Vaart et al., 2018) for ethical reasons it was difficult to discriminate on the grounds of psychological aspects. Therefore, lastly, but perhaps most noteworthy, the limitation is that mental health and depressive symptoms were not studied in the current paper and could be considered in future research.

A final limitation, particularly relevant to intervention studies in behavioral research, may be that much time has been spent with the experimental group, therefore, the results of this study can be attributed to the attention they received during the program. A comparative framework should be considered to provide an opportunity for the control group to receive equal attention (Aycock et al., 2018).

## Conclusion

Unemployment is considered one of the South African government’s top priorities (Ramaphosa, 2019). Although unemployment is a much-researched and timely topic, programs aimed at assisting the unemployed in South Africa often neglects important psychosocial aspects. Considering the severe repercussions of unemployment, an urgent need exists to intervene on a psychological level. Likewise, while derivatives of JOBS program have been successfully implemented in many countries, its exposure to circumstances and economic conditions similar to that of South Africa is limited. This is the first study to undertake a longitudinal analysis of the JOBS program within the South African context. The primary purpose of this paper was to implement the JOBS program in two low-income communities in South Africa.

The present study contributed to existing literature to support the effectiveness of the JOBS program in a variety of contexts. Overall, the Qhubekela Phambili program provided evidence to demonstrate the programs’ ability to enhance the job-search self-efficacy and the self-esteem of participants. Contrary to expectations, the program did not succeed in decreasing the levels of amotivation experienced by participants, which may reveal another valuable finding. Since the amotivated believe that their actions will not yield desired outcomes (Vansteenkiste et al., 2004) emphasis should be on helping the unemployed overcome such perceptions. Fostering beliefs of personal control may contribute significantly to coping with and overcoming unemployment.

## Author’s Note

This manuscript has been released in a repository at https://repository.nwu.ac.za/handle/10394/33069, Paver (2019).

## Data Availability Statement

The datasets generated for this study are available on request to the corresponding author.

## Ethics Statement

The studies involving human participants were reviewed and approved by the North-West University – Humanities and Health Research Ethics Committee (HHREC; NWU-HS-2018-0006). The participants provided their written informed consent to participate in this study.

## Author Contributions

This publication was based on the Ph.D. thesis of RP. HD, SR, and AV were co-authors as well as supervisors of the project. RB made conceptual contributions to the manuscript. All authors contributed to the article and approved the submitted version.

## Conflict of Interest

The authors declare that the research was conducted in the absence of any commercial or financial relationships that could be construed as a potential conflict of interest.

## References

[B1] Aycock D. M. Hayat M. J. Helvig A. Dunbar S. B. Clark P. C. (2018). Essential considerations in developing attention control groups in behavioral research. *Res. Nurs. Health* 41 320–328. 10.1002/nur.21870 29906317

[B2] Bandura A. (1986). The explanatory and predictive scope of self-efficacy theory. *J. Soc. Clin. Psychol.* 4 359–373. 10.1521/jscp.1986.4.3.359

[B3] Barry M. Reynolds C. Sheridan A. Egenton R. (2006). Implementation of the JOBS program in Ireland. *J. Public Mental Health* 5 10–25. 10.1108/17465729200600028

[B4] Brenninkmeijer V. Blonk R. W. (2011). The effectiveness of the JOBS program among the long-term unemployed: a randomized experiment in the Netherlands. *Health Promot. Int.* 27 220–229. 10.1177/1069072716657534 21653628

[B5] Caplan R. D. Vinokur A. D. Price R. H. (1997). “From job loss to reemployment: field experiments in prevention-focused coping,” in *Primary Prevention Works: Issues in Children’s and Families’ Lives*, eds Albee G. W. Gullotta T. P. (Thousand Oaks, CA: Sage), 341–379.

[B6] Caplan R. D. Vinokur A. D. Price R. H. Van Ryn M. (1989). Job seeking, reemployment, and mental health: a randomized field experiment in coping with job loss. *J. Appl. Psychol.* 74 759–769. 10.1037/0021-9010.74.5.759 2793774

[B7] Cheung F. M. Van de Vijver F. J. R. Leong F. T. L. (2011). Toward a new approach to the study of personality in culture. *Am. Psychol.* 66 593–603. 10.1037/a0022389 21261408

[B8] Choi J. N. Price R. H. Vinokur A. D. (2003). Self-efficacy changes in groups: effects of diversity, leadership, and group climate. *J. Organ. Behav.* 24 357–372. 10.1002/job.195

[B9] City of Johannesburg (2018). *Integrated Development Plan: 2018/19 Review.* Johannesburg: City of Johannesburg Metropolitan Municipality.

[B10] Clark L. A. Watson D. (1995). Constructing validity: basic issues in objective scale development. *Psychol. Assess.* 7 309–319. 10.1037/pas0000626 PMC6754793 30896212

[B11] Cobb-Clark D. A. (2015). Locus of control and the labor market. *IZA J. Lab. Econ.* 4 1–19. 10.1186/s40172-014-0017-x

[B12] Cohen J. (1988). *Statistical Power Analysis for the Behavioral Sciences*, 2nd Edn Hillsdale, NJ: Erlbaum.

[B13] Curran J. Wishart P. Gingrich J. (1999). *JOBS: A Manual for Teaching People Successful Job Search Strategies.* Michigan: University of Michigan.

[B14] Deci E. L. Ryan R. M. (1985). *Intrinsic Motivation and Self-Determination in Human Behavior.* New York, NY: Plenum.

[B15] Donaldson S. I. Weiss R. (1998). “Health, well-being, and organizational effectiveness in the virtual workplace,” in *The Virtual Workplace*, eds Igbaria M. Tan M. (Hershey, PA: Idea Group Publishing), 24–44.

[B16] Du Toit M. De Witte H. Rothmann S. Van den Broeck A. (2018). Unemployment experiences in context: a phenomenological study in two townships in South Africa. *J. Psychol. Africa* 28 122–127. 10.1080/14330237.2018.1454575

[B17] Field A. (2013). *Discovering Statistics Using SPSS.* Thousand Oaks, CA: SAGE Publications.

[B18] Hobfoll S. E. (1989). Conservation of resources: a new attempt at conceptualizing stress. *Am. Psychol.* 44 513–524. 2648906 10.1037//0003-066x.44.3.513

[B19] Houssemand C. Meyers R. Pignault A. (2019). Adaptation and validation of the perceived control in unemployment scale. *Front. Psychol.* 10:383. 10.3389/fpsyg.2019.00383 30873084 PMC6403124

[B20] IBM Corp (2017). *IBM SPSS Statistics for Windows, Version 25.0.* Armonk, NY: IBM Corp.

[B21] Jahoda M. (1982). *Employment and Unemployment: A Social-Psychological Analysis.* London: Cambridge University Press.

[B22] Kanfer R. Wanberg C. R. Kantrowitz T. M. (2001). Job search and employment: a personality–motivational analysis and meta-analytic review. *J. Appl. Psychol.* 86 837–855. 10.1037/0021-9010.86.5.837 11596801

[B23] Kluve J. Puerto S. Robalino D. Romero J. M. Rother F. Stöterau J. (2017). Interventions to improve the labour market outcomes of youth: a systematic review of training, entrepreneurship promotion, employment services, and subsidized employment interventions. *Campbell Syst. Rev.* 12 1–288. 10.4073/csr.2017.12

[B24] Lefcourt H. M. (2014). *Locus of Control: Current Trends in Theory and Research.* New York, NY: Psychology Press.

[B25] Liu S. Huang J. L. Wang M. (2014). Effectiveness of job search interventions: a meta-analytic review. *Psychol. Bull.* 140 1009–1041. 10.1037/a0035923 24588365

[B26] Malmberg-Heimonen I. E. West B. T. Vuori J. (2019). Long-term effects of research-based and practice-based job search interventions: an RCT reevaluation. *Res. Soc. Work Pract.* 29 36–48. 10.1177/1049731517748424

[B27] McKee-Ryan F. M. Song Z. Wanberg R. Kinicki A. J. (2005). Psychological and physical well-being during unemployment: a meta-analytic study. *J. Appl. Psychol.* 90 53–74. 10.1037/0021-9010.90.1.53 15641890

[B28] Michigan Prevention Research Center (2013). *The JOBS Project for the Unemployed: Update.* Michigan: Michigan Prevention Research Center.

[B29] Møller V. (1991). *The Unemployment Blues: Psychological Effects of Unemployment on the Individual. (Working Paper No.* 6). Durban: University of Natal.

[B30] Paul K. I. Moser K. (2009). Unemployment impairs mental health: meta-analyses. *J. Vocat. Behav.* 74 264–282. 10.1016/j.jvb.2009.01.001

[B31] Paver R. (2019). *Vocational Interventions for the Unemployed.* Doctoral dissertation, North-West University, Vanderbijlpark.

[B32] Paver R. Blonk R. W. B. Rothmann S. De Witte W. Van den Broeck A. (2018). *Qhubekela Phambili Career-enhancement Programme: AJobseeker’s Guide to Employment*. Vanderbijlpark, South Africa: North-West University.

[B33] Paver R. De Witte W. Rothmann S. Van den Broeck A. Blonk R. W. B. (2019a). A systematic literature review of the implementation and evaluation of the JOBS program: a suggested framework for South Africa. *South Afr. J Econ. Manag. Sci.* 23:a3049.

[B34] Paver R. Rothmann S. Van den Broeck A. De Witte W. (2019b). A review of labour market interventions to assist the unemployed in two townships in South Africa. *South Afr. J. Econ. Manag. Sci.* 45:a1596 10.4102/sajip.v45i0.1596

[B35] Preuss M. Hennecke J. (2018). Biased by success and failure: how unemployment shapes locus of control. *Lab. Econ.* 53 63–74.

[B36] Price R. H. Choi J. Vinokur A. D. (2002a). Links in the chain of adversity following job loss: how economic hardship and loss of personal control lead to depression, impaired functioning and poor health. *J. Occupat. Health Psychol.* 7 302–312. 10.1037//1076-8998.7.4.302 12396064

[B37] Price R. H. Vinokur A. D. Friedland D. S. (2002b). “The job seeker role as resource in achieving reemployment and enhancing mental health new directions,” in *Socioeconomic Conditions, Stress and Mental Health Disorders: Toward a New Synthesis of Research and Public Policy*, eds Maney A. Ramos J. (Washington, DC: National Institute of Mental Health).

[B38] Price R. H. Fang L. (2002). Unemployed Chinese workers: the survivors, the worried young and the discouraged old. *Int. J. Hum. Resour. Manag.* 13 416–430. 10.1080/09585190110111459

[B39] Price R. H. Van Ryn M. Vinokur A. D. (1992). Impact of a preventive job search intervention on the likelihood of depression among the unemployed. *J. Health Soc. Behav.* 33 158–167. 10.2307/2137253 1619263

[B40] Price R. H. Vinokur A. D. (1995). “Supporting career transitions in a time of organizational downsizing: the Michigan JOBS program,” in *Employees, Careers and Jobs Creation: Developing Growth-Orientated Human Resource Strategies and Programs*, ed. London M. (San Francisco, CA: Jossey- Bass Publishers).

[B41] Price R. H. Vinokur A. D. (2014). “The JOBS program: impact on job seeker motivation reemployment, and mental health,” in *Oxford Handbook of Job Loss and Job Search*, eds Klehe U. van Hooft E. A. J. (Oxford: Oxford University Press).

[B42] Ramaphosa C. (2019). *State of the Nation Address of the President of South Africa, Cyril Ramaphosa.* Cape Town: Houses of Parliament.

[B43] Rosenberg M. (1965). *Society and the Adolescent Self-Image.* Princeton, NJ: Princeton University Press.

[B44] Rosenberg M. Schooler C. Schoenbach C. Rosenberg F. (1995). Global self-esteem and specific self-esteem: different concepts, different outcomes. *Am. Sociol. Rev.* 60 141–156. 10.2307/2096350

[B45] Rotter J. B. (1966). Generalized expectancies for internal versus external control of reinforcement. *Psychol. Monogr.* 80 1–28. 10.1037/h0092976 5340840

[B46] Ryan R. M. Deci E. L. (2000). Intrinsic and extrinsic motivations: classic definitions and new directions. *Contemp. Educ. Psychol.* 25 54–67. 10.1006/ceps.1999.1020 10620381

[B47] Saks A. M. Ashforth B. E. (1999). Effects of individual differences and job search behaviors on the employment status of recent university graduates. *J. Vocat. Behav.* 54 335–349. 10.1006/jvbe.1998.1665

[B48] Schaufeli W. B. VanYperen N. W. (1993). Success and failure in the labour market. *J. Organ. Behav.* 14 559–572.

[B49] Shirom A. Vinokur A. Price R. (2008). Self-efficacy as a moderator of the effects of job-search workshops on re-employment: a field experiment. *J. Appl. Soc. Psychol.* 38 1778–1804. 10.1111/j.1559-1816.2008.00369.x

[B50] Statistics South Africa (2018). *Quarterly labour force survey: Quarter 3, 2018.* Pretoria: Statistics South Africa.

[B51] Van den Hof R. A. (2015). *Self-esteem, self-efficacy and employability among disadvantaged youth in Orange Farm, Johannesburg.* Unpublished master’s thesis, University of Johannesburg, Johannesburg.

[B52] Van der Vaart L. De Witte H. Van den Broeck A. Rothmann S. (2018). A psychosocial typology of the unemployed in South Africa. *South Afr. J. Psychol.* 48 179–192. 10.1177/0081246317721600

[B53] Van der Vaart L. Van den Broeck A. Rothmann S. Witte H. D. (2019). Experiences, attitudes, and behaviors of the unemployed: the role of motivation and psychological needs. *Psychol. Rep.* 10.1177/0033294119849020 [Epub ahead of print]. 31094660

[B54] Van Ryn M. Vinokur A. D. (1992). How did it work? An examination of the mechanisms through which an intervention for the unemployed promoted job-search behavior. *Am. J. Community Psychol.* 20 577–597. 10.1007/BF00941773 1485612

[B55] Vansteenkiste M. Lens W. De Witte H. Feather N. T. (2005). Understanding unemployed people’s job search behaviour, unemployment experience and well-being: a comparison of expectancy-value theory and self-determination theory. *Br. J. Soc. Psychol.* 44 268–287. 10.1348/014466604X17641 16004649

[B56] Vansteenkiste M. Lens W. De Witte S. De Witte H. Deci E. L. (2004). The ‘why’ and ‘why not’ of job search behaviour: their relation to searching, unemployment experience, and well-being. *Eur. J. Soc. Psychol.* 34 345–363. 10.1002/ejsp.202

[B57] Vinokur A. Caplan R. D. (1987). Attitudes and social support: determinants of job-seeking behavior and well-being among the unemployed. *J. Appl. Soc. Psychol.* 17 1007–1024. 10.1111/j.1559-1816.1987.tb02345.x

[B58] Vinokur A. D. Price R. H. (2015). “Promoting reemployment and mental health among the unemployed,” in *Sustainable Working Lives. Aligning Perspectives on Health, Safety and Well-Being*, eds Vuori J. Blonk R. Price R. (Dordrecht: Springer), 171–186. 10.1007/978-94-017-9798-6_10

[B59] Vinokur A. D. Price R. H. Caplan R. D. (1991a). From field experiments to program implementation: assessing the potential outcomes of an experimental intervention program for unemployed persons. *Am. J. Community Psychol.* 19 543–562. 10.1007/978-1-4615-0565-5_3 1755435

[B60] Vinokur A. D. Van Ryn M. Gramlich E. M. Price R. H. (1991b). Long-term follow-up and benefit-cost analysis of the Jobs program: a preventive intervention for the unemployed. *J. Appl. Psychol.* 76 213–219. 1905293 10.1037/0021-9010.76.2.213

[B61] Vinokur A. D. Price R. H. Caplan R. D. (1996). Hard times and hurtful partners: how financial strain affects depression and relationship satisfaction of unemployed persons and their spouses. *J. Pers. Soc. Psychol.* 71 166–179. 10.1037/0022-3514.71.1.166 8708998

[B62] Vinokur A. D. Price R. H. Caplan R. D. Van Ryn M. Curran J. (1995). “The Jobs I preventive intervention for unemployed individuals: short-and long-term effects on reemployment and mental health,” in *Job Stress Interventions*, eds Murphy L. R. Hurrell J. J. Jr. Sauter S. L. Keita G. P. (Washington, DC: American Psychological Association), 125–138. 10.1037/10183-009

[B63] Vinokur A. D. Schul Y. (1997). Mastery and inoculation against setbacks as active ingredients in the JOBS intervention for the unemployed. *J. Consult. Clin. Psychol.* 65 867–877. 10.1037/0022-006X.65.5.867 9337505

[B64] Vinokur A. D. Schul Y. (2002). The web of coping resources and pathways to reemployment following a job loss. *J. Occup. Health Psychol.* 7 68–83. 10.1037/1076-8998.7.1.68 11827235

[B65] Vinokur A. D. Schul Y. Vuori J. Price R. H. (2000). Two years after a job loss: long term impact of the JOBS program on reemployment and mental health. *J. Occup. Health Psychol.* 5 32–47. 10.1037/1076-8998.5.1.32 10658883

[B66] Vinokur A. D. Van Ryn M. (1993). Social support and undermining in close relationships: their independent effects on the mental health of unemployed persons. *J. Pers. Soc. Psychol.* 65 350–359. 8366424 10.1037//0022-3514.65.2.350

[B67] Vuori J. Silvonen J. (2005). The benefits of a preventive job search program on re-employment and mental health at 2-year follow-up. *J. Occup. Organ. Psychol.* 78 43–52. 10.1348/096317904X23790 30467716

[B68] Vuori J. Silvonen J. Vinokur A. D. Price R. H. (2002). The Työhön Job Search Program in Finland: benefits for the unemployed with risk of depression or discouragement. *J. Occup. Health Psychol.* 7 5–19. 10.1037/1076-8998.7.1.5 11827233

[B69] Wanberg C. R. Ali A. A. Csillag B. (2020). Job seeking: the process and experience of looking for a job. *Ann. Rev. Organ. Psychol. Organ. Behav.* 7 315–337.

[B70] World Bank (2018). *Overcoming Poverty and Inequality in South Africa: An Assessment of Drivers.* Washington, DC: World Bank.

