# Supplementary material for: The Implementation and Evaluation of the South African Adaptation of the JOBS Program
Source: Front Psychol. 2020 Jul 14;11:1418. doi: 10.3389/fpsyg.2020.01418 (PMC7371856; doi:10.3389/fpsyg.2020.01418)
Supplement: Supplementary file 1 [file Presentation_1.pdf]

OPTENTIA vliruos

Qhubekela Phambili

Qhubekela Phambili Career-enhancement Programme

A Jobseeker's Guide to Employment

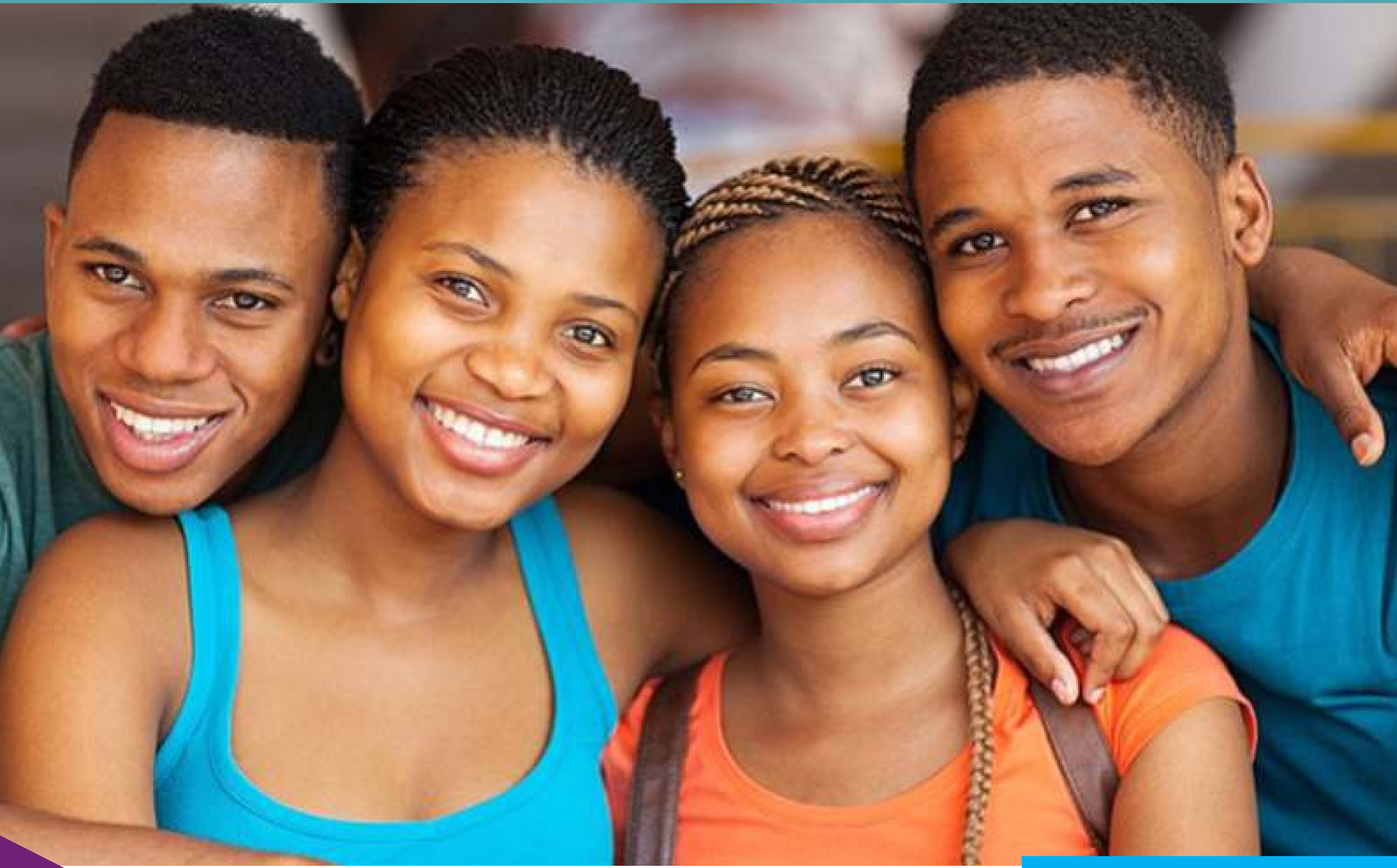

Name:

KU LEUVEN

NWU®
NORTH-WEST UNIVERSITY
NOORDWES-UNIVERSITEIT
UNIBESITI YA BOKONE-BOPHIRIMA

Optimising potential

Welcome note

The world is full of exciting opportunities for people who are prepared to think out of the box, can adapt to change, and are prepared to keep on developing themselves. We also know that our world presents challenges, many of which we think we have no control over. The Jobseeker's Guide to Employment provides you with a backpack filled with resources that will help guide your journey. Insights, such as your skills, values, passions and dreams are explored during this workshop, while simultaneously advice to make better career choices, to prepare you for job applications, and to deal with job interviews (and possible setbacks) in the changing world of work is also provided. In your backpack, you will also find information about great people in your community who can help you cope with unemployment in one way or another. We hope that you will find these resources valuable.

Of course, you can also visit the Optentia website (www.optentia.co.za), where you can find out more about the latest research on unemployment, ideas on how to cope with unemployment, and useful resources in your community. On our website, we provide extra information if you want to read more about the content covered in this guide.

We hope that you will use and enjoy the guide!

Qhubekela Phambili Research Team

Road map

Welcome note	2
Necessities for your backpack	6
What you will learn	7
About me	8
About my partner	9
What I stand for	10
Skills	11
Personal characteristics	12
Transferable skills	13
My experiences per day	14
My passions	15
Positive people who have had an influence on my life	17
What I find important in my work	18
Exploring possible careers based on my attributes	19
Possible careers and requirements	20
The time machine - interview	21
Developing my career	22
Action plan for the future	23
Discovering my network	25
A map of my own network	26
My network plan	27
Additional resource: Asking for job leads/training opportunities	28
Additional resource: Steps in contacting people for job leads/training opportunities	29
Additional resource: Asking for job leads/training opportunities	30
Additional resource: Employment opportunities	31

Additional resource: Self-employment opportunities 33
- Development finance institutions (DFIs) 34
- Other 35
Additional resource: Education and training 36
- Universities 36
- Universities of technology 36
- Sector education and training authorities (SETAs) 37
- Other 38
Additional resource: Financial assistance and incentive schemes 39
- Incentive schemes 40
- Industrial development-related incentives 41
- Production incentive (PI) 42
Additional resource: Career services 44
- National Youth Development Agency (NYDA) 44
- NYDA Youth Advisory Centres 45
- Thusong Service Centres 45
Additional resource: Support institutions 46
Ways to present myself 47
Tips for good listening, speaking, and writing 48
Tips for effective communication 49
Observing and giving feedback 50
Role play 51
Facing my fears 52
Additional resource: The ultimate marketing tool: your CV 54
Additional resource: Checklist for CVs 55
Additional resource: Example of a CV 56

Additional resource: Tips for making your list of job references 60
Additional resource: Suggestions for writing a cover letter 61
Additional resource: Example of a cover letter 62
Additional resource: Informational interviewing 63
Additional resource: Tips for conducting an informational interview 64
Additional resource: Pre-interview checklist 65
Additional resource: Post-interview checklist 66
Additional resource: Follow-up plan 67
Additional resource: Dressing for the interview 68
Additional resource: Organising your job search 69
Additional resource: Social media for professionals 72
Additional resource: Tips for completing documents/forms adequately 73
I commit myself to … 74
Final statement 75
What I have learnt from …/What I admire about … 76
What I want to let go 77
A thousand-step journey begins with a single step! 78
Some final thoughts 79
References 80

Necessities for your backpack

The basics

- Energy/enthusiasm
- Positive attitude
- Active participation
- Commitment
- Respect for one another

Each person has a unique journey, and therefore, it is important to know where you are headed. Choosing a career (or starting your own business) may be one of the most important choices you will make in your life. With this workshop, we hope that you will gain some insight into these major decisions. For this reason, we strongly advise that you make the most of this opportunity and take as much from it as you can.

Your journey requires equipment that differs from that required by others. It is important to determine what skills, qualifications, and experience (if necessary) are needed to embark on your journey. Finding ways, identifying resources, making an action plan, and setting deadlines are crucial to achieve the goals of your unique journey.

1.1 What you will learn

Session 1

- Getting to know one another
- Discovering your skills, strengths, and traits
- Identifying how these skills, strengths, and traits can be used in multiple positions/professions
- Exploring and sharing your values, interests, and hobbies with others

Session 2

- Discovering what your passions and values are
- Exploring your dream job
- Linking your skills, passions, values, and dreams to your career
- Discovering what functions, professions, and training suit you
- Forming a career image

Session 3

- Knowing better what you want to learn and what you want to achieve in the future
- Making an action plan and setting goals to achieve your vision of the future

Session 4

- Mapping your network and exploring its usefulness
- Other valuable resources

Session 5

- CV, cover letter, and interview tips
- Effective communication skills

What would you like to learn?

1.2 About me

Answeer the following questions about yourself. Then find out the following about your partner. Then switch roles, and your partner will find out about you.

Later, you will have an opportunity to introduce each other to the group.

Name: ....

Age: ....

Experience (work, training, education):

....

....

....

The job that I would like to do:

....

....

....

Something special or interesting about me:

....

....

....

What I want to learn from the workshop:

....

....

....

1.3 About my partner

Find out the following about your partner.

Name: ....

Age: ....

Experience (work, training, education):

....

....

....

The job that he/she would like to do:

....

....

....

Something special or interesting about him/her:

....

....

....

What he/she wants to learn from the workshop:

....

....

....

1.4 What I stand for

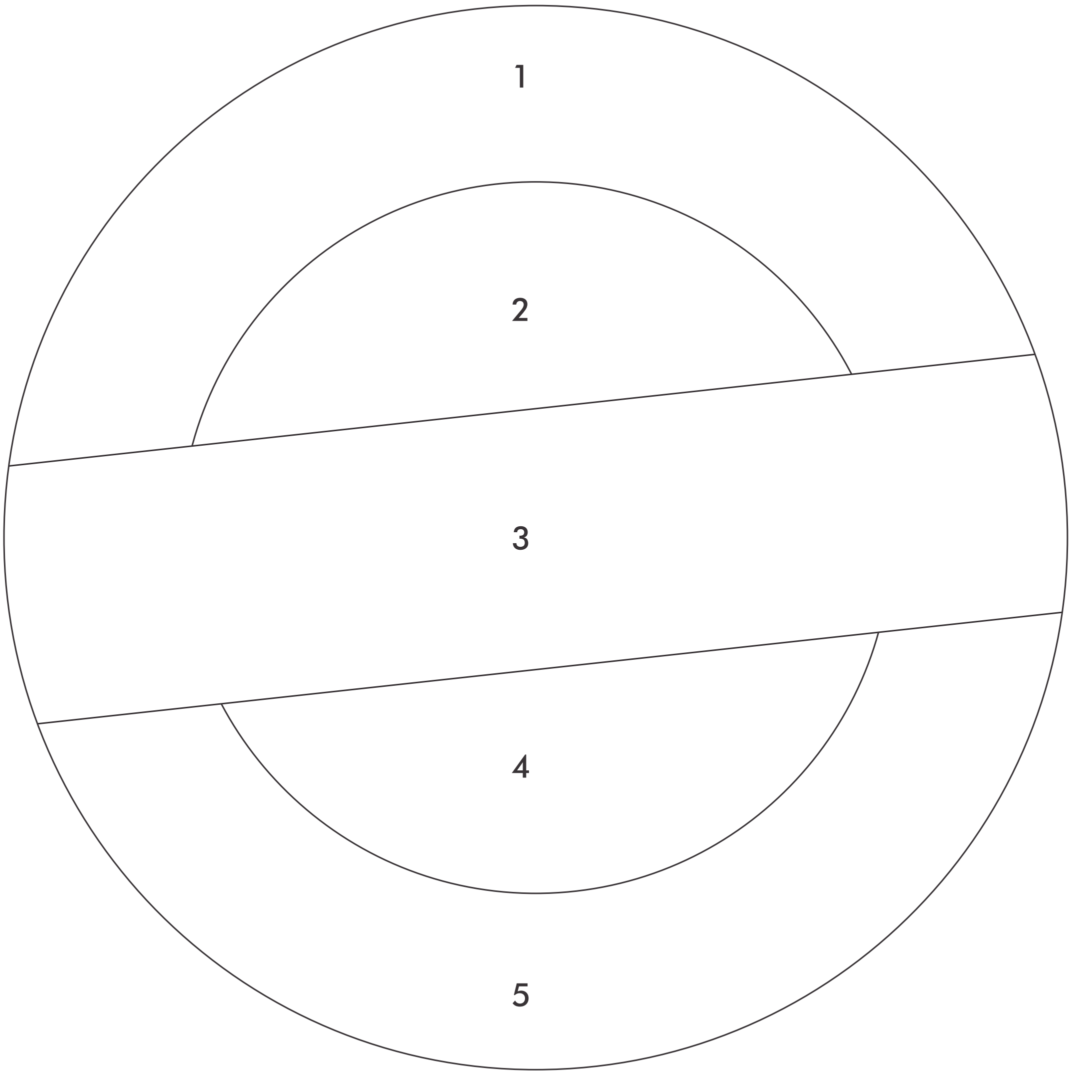

Explanation of the numbers:
Area 1 = Draw or write down something at which you excel.
Area 2 = Draw something you are proud of when you have achieved it. Area 3 = Draw or write down what you stand for in your life.
Area 4 = Draw something that you prefer to do in your spare time.
Area 5 = Draw or write down what your "hidden" qualities / Something special or interesting about yourself.

1.5 Skills

Skills are things that you can do (well). Skills can be learnt or developed in all kinds of situations, such as at home, in your hobbies, at school, or at work. Below are a number of skills. If you finish the sentence “I can … well”, that is often called a skill. In the list below, check (√) all the characteristics you have.

Collaborating with others	Working with your head	Working with your body
[ ] Listen	[ ] Analyse data or facts	[ ] Assemble things
[ ] Supervise	[ ] Investigate	[ ] Build
[ ] Manage	[ ] Audit	[ ] Inspect
[ ] Administer	[ ] Calculate	[ ] Construct
[ ] Teach	[ ] Budget	[ ] Repair equipment
[ ] Interview	[ ] Handle money	[ ] Repair buildings
[ ] Negotiate	[ ] Keep financial records	[ ] Operate equipment
[ ] Mediate	[ ] Organise	[ ] Operate machinery
[ ] Lead	[ ] Evaluate	[ ] Drive
[ ] Motivate	[ ] Research	[ ] Work with your hands
[ ] Make decisions	[ ] Compare	[ ] Handle precision skills
[ ] Facilitate	[ ] Count	[ ] Have computer skills
[ ] Direct	[ ] Classify information	
[ ] Explain	[ ] Be detail oriented	[ ] Other
[ ] Plan	[ ] Find information	....
[ ] Coordinate	[ ] Inspect	
[ ] Delegate	[ ] Observe	**Creative/artistic skills**
[ ] Communicate verbally	[ ] Copy	[ ] Paint
[ ] Correspond	[ ] Edit	[ ] Do graphic design
[ ] Speak in public	[ ] Write	[ ] Dance
[ ] Counsel	[ ] Measure	[ ] Do creative movement
[ ] Coach	[ ] Translate	[ ] Sing
[ ] Cooperate		[ ] Draw
[ ] Encourage	[ ] Other	[ ] Act
[ ] Inspire	....	[ ] Play a musical instrument
[ ] Persuade	....	[ ] Compose music
[ ] Sell	....	[ ] Mime
[ ] Other		[ ] Do creative writing
....		[ ] Take photos
....		[ ] Bake/cook
....		[ ] Other
		....

1.6 Personal characteristics

Characteristics are things that are really unique to you and that you take with you in all situations. If you finish the sentence “I am ...”, that is often called a trait. Traits relate to the way you do things and how you prepare yourself. For example, traitsto whichemployers attach great importance are being trustworthy, nice, and honest. You can show and use most characteristics in different situations. However, some characteristics are more important in a certain situation than in other situations. For example, reliability is especially important when you work with money.

In the list below, check (√) all the characteristics you have.

[ ] Honest
[ ] Reliable
[ ] Quiet/calm
[ ] Motivated
[ ] Friendly
[ ] Cheerful
[ ] Curious
[ ] Flexible
[ ] Helpful
[ ] Creative
[ ] Independent
[ ] Respectful
[ ] Caring
[ ] Open
[ ] Attentive
[ ] Accurate
[ ] Brave
[ ] Energetic
[ ] Enthusiastic
[ ] Practical
[ ] Open-minded
[ ] Sincere
[ ] Optimistic
[ ] Steadfast
[ ] Positive thinker
[ ] Patient
[ ] Physically strong
[ ] Self-assured
[ ] Funny

[ ] Responsible
[ ] Tactful
[ ] Persevering
[ ] Hard-working
[ ] Conscientious
[ ] Decisive
[ ] Intuitive
[ ] Pleasant
[ ] Understanding
[ ] Cosy
[ ] Smart/intelligent
[ ] Tolerant
[ ] Deliberate/thoughtful
[ ] Sweet
[ ] Involved with others
[ ] Serious
[ ] Driven
[ ] Enterprising
[ ] Confident
[ ] Social
[ ] A go-getter

[ ] Other

____
____
____
____

1.7 Transferable skills

You may learn general skills in a certain situation, but you can use them in all kinds of other situations. You can "take" them from one situation to another. In the list below, check (✓) all the skills you have. Go through the list again, and put an asterisk (*) after all the skills you would like to use. You may also add other skills to the list.

[ ] Adapt easily
[ ] Work independently
[ ] Be a caretaker
[ ] Organise activities
[ ] Generate ideas
[ ] Invent solutions
[ ] Convince others
[ ] Do calculations
[ ] Plan activities
[ ] Stay calm
[ ] Learn quickly
[ ] Work with others
[ ] Work with a computer
[ ] Remember information
[ ] Stand up for myself
[ ] Solve problems
[ ] Create a good atmosphere
[ ] Take responsibility
[ ] Repair things
[ ] Sell things
[ ] Follow instructions

[ ] Offer service
[ ] Work fast
[ ] Lead
[ ] Listen
[ ] Assemble objects
[ ] Deal with money
[ ] Explain/teach something
[ ] Put others at ease
[ ] Organise information
[ ] Deal with animals
[ ] Type
[ ] Work hard
[ ] Take decisions
[ ] Write

[ ] Other

____
____
____
____

I

1.8 My experiences per day

Every day, write down what you liked and found useful in the training, what you are good at, what you are proud of, or what you have learnt. What ideas have you gained to keep developing yourself in your career?

	What did you like and find useful?<br>What are you good at or proud of?<br>What did you learn?	Ideas to keep developing yourself in your career
Day 1		
Day 2		
Day 3		
Day 4		
Day 5		
Day 6		

2.1 My passions

Passions are activities and things that make you very enthusiastic and that you enjoy very much. Describe your greatest passion(s) below. You can use the questions provided to guide you.

What things do I get excited about?

....

....

When am I at my best?

....

....

What do I get a kick out of?

....

....

What do I really like?

....

....

When does time fly by?

....

....

What are some childhood dreams or interests you were never able to fully explore, but still find intriguing?

....

....

Who is someone in your life or in history whose life and work inspire and excite you? Why?

……………………………………………………………………………………………………

……………………………………………………………………………………………………

What specific activities/hobbies have you had in the past that you really enjoyed and found engaging?

……………………………………………………………………………………………………

……………………………………………………………………………………………………

What would your ideal day look like? Describe it in detail from morning until bedtime.

……………………………………………………………………………………………………

……………………………………………………………………………………………………

What is something (or several things) you would really like to achieve before you die?

……………………………………………………………………………………………………

……………………………………………………………………………………………………

What would make you feel proud of yourself?

……………………………………………………………………………………………………

……………………………………………………………………………………………………

If you could start afresh all over again with your life and career, what would you do differently?

……………………………………………………………………………………………………

……………………………………………………………………………………………………

2.2 Positive people who have had an influence on my life

Make a list of positive people who are important in your life. These may be people you know personally (such as family, friends, or acquaintances) or people you do not know personally, but for whom you have great appreciation (such as movie stars, politicians, sports figures, spiritual leaders, etc.). They are people you admire and see as an example in your life.

Name of person	Reason

2.3 What I find important in my work

Below, there are two statements in each row. Select the statement that suits your preferences best. Which would you find more important in your career?
Usually I prefer...

	Working on my own		Collaborating with colleagues
	A lot of freedom, a little guidance		A lot of clarity, a lot of guidance
	Short periods of concentration		Long periods of concentration
	Doing multiple things at once		Doing one thing at a time
	A slow and quiet work schedule		A fast and busy work schedule
	Spontaneous actions		Planning, thinking ahead
	Making decisions quickly		Thinking about decisions
	Working in a busy environment		Working in a quiet environment
	Working with my hands		Working with my head (thinking)
	Working outside		Working inside
	Alternating working hours		Fixed working hours
	Routine in my work		A lot of variety in my work
	Working during the day		Working in the evening
	Earning a lot of money		Having fun and interesting work
	Other		

2.4 Exploring possible careers based on my attributes and available opportunities

Possible career: ....

Skills: ....

Personal characteristics: ....

Passions: ....

Values: ....

Possible career: ....

Skills: ....

Personal characteristics: ....

Passions: ....

Values: ....

Possible career: ....

Skills: ....

Personal characteristics: ....

Passions: ....

Values: ....

3.1 Possible careers applicable to me

Opportunity:	Requirements
1	• • •
2	• • •
3	• • •
4	• • •
5	• • •

3.2 The time machine – interview

Imagine that you are looking back on your career in five years' time. What does it look like? What have you done and achieved in this period from today onwards? Write down what your career looks like in terms of the following:

Work (what job or profession do you have? Have you had another job or profession in the interim?)

………………………………………………………………………………………………

………………………………………………………………………………………………

Skills (what skills did you develop / What training / courses did you do and which ones will you possibly still follow?)

………………………………………………………………………………………………

………………………………………………………………………………………………

Experience (how did you get the necessary experience?)

………………………………………………………………………………………………

………………………………………………………………………………………………

Network (who helped you?)

………………………………………………………………………………………………

………………………………………………………………………………………………

Other possibilities for work (which possible functions or professions do you see for yourself?)

………………………………………………………………………………………………

………………………………………………………………………………………………

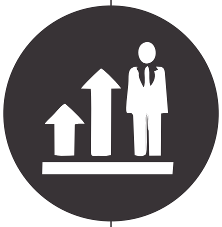

3.3 Developing my career

Focussing on your own skills, qualifications, and experience, answer the questions below.

What skills, qualifications, experience, etc. do I have?

....

....

....

....

....

....

What do I need to obtain the job/self-employment opportunity I want to have?

....

....

....

....

....

....

What are my options to gain what I require?

....

....

....

....

....

....

3.4 Action plan for the future

My vision for the future – the goal I want to work towards is:

....

....

....

My short-term (next week to one month) goals are:

....

....

....

For these, I will do the following:

Action ....

Action ....

Action ....

Action ....

My medium-term (next year) goals are:

....

....

....

For these, I will do the following:

Action ....

Action ....

Action ....

Action ....

My longer-term (next five years) goals are:

....

....

....

For these, I will do the following:

Action ....

Action ....

Action ....

Action ....

4.1 Discovering my network

Research has found that approximately 63% of all jobs people find are through contacts among people. This means that a large proportion of people get a job via a network! Your network may be even more important than responding to job advertisements and sending your CV (curriculum vitae). Family members, friends, acquaintances, and (former) colleagues can give you information about vacancies and jobs. Of course, you can also approach these people about training and/or internships. It is important that you ask them whether they know anything about the training or job for which you are looking. Maybe they know people who know more about it. You can also ask them to keep an eye on vacancies and keep you informed about important information on courses and jobs. Becoming aware of your network and understanding how you can use it are important for your career!

~~~~~~

Your own social network may well be your best way of searching for education or work!

~~~~~~

Most people know a lot more people than they initially think. For example, you can think of the following people:

Family members	(Ex-)colleagues and fellow students	Friends and acquaintances
• Mother<br>• Father<br>• Brother(s)<br>• Sister(s)<br>• Your partner<br>• Uncles and aunts<br>• Nephews and nieces<br>• In-laws<br>• Grandma<br>• Grandpa	• From your previous job(s)<br>• From your own department<br>• From other departments<br>• With whom you went to lunch<br>• Who said goodbye to you when you left your previous job<br>• Who have left the organisation<br>• With whom you had conversations during social gatherings	• Current neighbours<br>• Previous neighbours<br>• Shop clerks<br>• Doctors<br>• Hairdressers<br>• Bus/taxi drivers<br>• Postmen<br>• Members of a club<br>• School friends<br>• Neighbourhood friends<br>• Acquaintances of friends<br>• Teachers<br>• Priest/pastor<br>• Church members<br>• Consultant(s)

4.2 A map of my own network

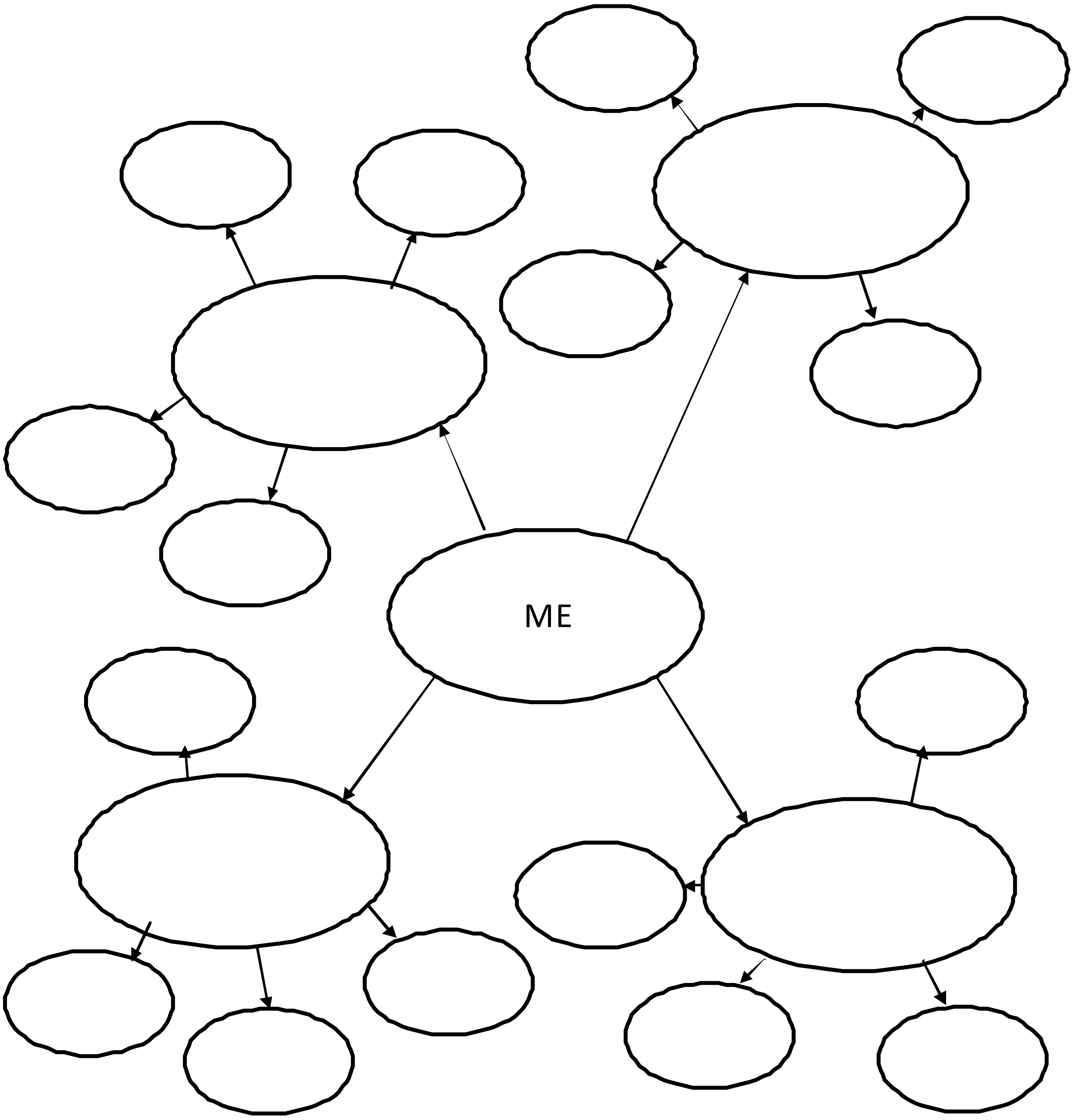

4.3 My network plan

(For what purpose will I approach whom, how, and when?)

What do I want to achieve?

Goal:

Whom am I going to approach for this (via whom)?

Person:

How do I approach this person?

How:

When will I approach this person?

When:

Additional resource: Asking for job leads/ training opportunities

C: Client A: Applicant

A: (This contact is a former boss from a summer job.) Hello, Mr Radebe. This is ________________. Do you have a moment?

C: Yes, what's on your mind?

A: I've been hunting for a different type of job from the one I did for you last summer. I want to work in a framing shop and maybe learn some new skills. I'm good with my hands, and I thought this might be a way of combining something I like to do with a job.

C: Gee, I don't think our day care business has much to do with framing.

A: That's true, but I thought that maybe you come into contact with customers who might have that type of operation. Perhaps you might hear about an opening.

C: Well, we do serve clients from a wide range of backgrounds. I could keep my ears open.

A: I'd really appreciate that. Is there anyone else you know who might have some inside knowledge about such jobs?

C: My brother-in-law, Carl, is in the wood-supply business. He might know of something.

A: He sound like a great lead. Could I call him up, or could you ask him about it?

C: Sure, call him up.

A: Is it OK for me to tell him you suggested that I call him?

C: Of course. His number is ________________.

A: This is really helpful, Mr Radebe. May I check back with you in a week or so to see whether you have heard of any openings?

C: Sure.

A: Thanks, Mr Radebe. I really appreciate your help.

C: Glad to help. Good luck.

Additional resource: Steps in contacting people for job leads/training opportunities

Steps	What you could say
Greet the person, and give your name.	"Hi Mary. This is _________. How are you?"
Ask if he/she has a moment to talk.	"Do you have a few minutes to talk?"
Explain that you are calling to explore new job opportunities.	"Great! I wanted to let you know that I'm looking for a job…"
State the type of job in which you are interested.	"…where I can work with children."
Describe the experience you have that relates to the job you want. State your skills and experience in a positive manner.	"I've had lots of experience in taking care of children between the ages of two and five, and I also recently volunteered at a primary school aftercare centre."
Ask whether the person has information about job leads.	"Do you know of any job possibilities or openings in this area?"<br>"Do you know of anyone who might have some inside knowledge in this area or who works in this field?"
Ask whether it is OK to use his/her name when you make contact and if the person refers you to someone else.	"Thanks for the lead. Would it be all right if I use your name when I call ______?"
Thank the person. (A written "Thank you" note is a nice gesture for job-lead contacts who have been particularly helpful.)	"Thanks, you've been a great help! I really appreciate it."
Ask whether you can keep in touch.	"Will it be OK if I call you again in a week or two to see whether you've heard of any other openings?"

Additional resource: Asking for job leads/ training opportunities

1. List two people or places you could call and ask about job openings. (You are not committing yourself to calling anyone. You are just considering the possibility.)

   ____

   ____

2. Tell your partner the names of the people/places you could call.

3. Your partner will role-play these people/places. Practise asking your partner about job leads.

4. You might want to jot down a few reminders of what to say; or you might want to use the handout Steps in contacting people for job leads.

5. Your partner will give you feedback after each job-lead call you make.

6. When you have practised asking two people/places, switch roles, and your partner will practise calling two people/places.

Additional resource: Employment opportunities

Tshepo 1 Million

Opportunities that are available through Tshepo 1 Million:

- Formal employment in companies of all sizes
- Public works programmes
- Microenterprise opportunities
- Work experience and volunteering opportunities to build your CV
- Learning opportunities

Apply online: www.tshepo.mobi

Employment Services of South Africa (ESSA)

A service by the Department of Labour to help people to have a better chance of finding a job and skills development opportunities in the labour market. Employment Services of South Africa (ESSA) is a service for all South African people, aiming to provide benefits to work-seekers, employers, the youth, people with disabilities, and many other people. The employment services are provided free of charge, so you can only win by making use of them.

Register your CV online or at Department of Labour centres.

Refer to this link for further instructions: http://www.labour.gov.za/DOL/downloads/documents/useful-documents/skills-developmentact/Employment%20Services%20of%20South%20Africa%20pamphlet

Graduate Asset Programme

The Graduate Asset Programme (GAP) is an internship programme aimed at growing the SME sector in South Africa. By helping place thousands of capable and willing unemployed graduates in internships, GAP assists the host businesses to gain much-needed skills and the graduates to gain valuable, practical learning experience.

Website: www.gogap.co.za

JumpStart

JumpStart is an exciting work readiness programme that develops the skills of unemployed young people and links them to career opportunities in the retail sector and the supply chain.

Email: info@mrpfoundation.com
Website:
http://mrpfoundation.org/Programmes/jumpstart.aspx

NWU CoachLab® programme

The CoachLab® programme entails the following:

- A 12-month facilitated programme offered to postgraduate students in the ICT sector
- A project-based programme to complement the formal studies of the students
- A focus on developing leadership in the information and communications technology sector

- Development of emotional intelligence skills through soft skills training as well as coaching and mentoring
- Providing sponsoring partners with access to highly skilled human resources to assist in the development of new applications

Tel.: 016 910 3393
Email: bhive@nwu.ac.za
Website:
http://commerce.nwu.ac.za/bhive-edc/coachlabvaal

NWU bhive Enterprise Development Centre – Enterprising Women Programme
This is an eight-month programme, geared to provide business support services to women who want to establish their own businesses.

Tel.: 016 910 3393
Email: bhive@nwu.ac.za
Website: commerce.nwu.ac.za/bhive-edc/enterprsing-woman-programme
Apply online:
http://applications.incubatorportal.com

NWU – Green Learnership Programme
The programme is based on an "incubator model", providing unemployed matriculants and college graduates with the opportunity to gain meaningful workplace experience through a structured mentoring programme, which includes entrepreneurial, leadership, and soft skills.

Contact person: Elliot Mogosi
Tel.: 016 910 3599
Email: emogosi@theinnovationhub.com
Website:
http://commerce.nwu.ac.za/bhive-edc/green-learnership-programme

Additional resource: Self-employment opportunities

NYDA Entrepreneurship Development Programme (EDP)
The Entrepreneurship Development Programme is aimed at creating a conducive environment for young entrepreneurs to access relevant entrepreneurship skills, knowledge, values, and attitudes for their businesses.

Website:
www.nyda.gov.za/Entrepreneurship-Development-Programme/Pages/default.aspx

Small Enterprise Development Agency (SEDA)
SEDA was created to support the growth of small enterprises in South Africa, to help create a better regulatory environment for small enterprises, and to encourage a culture of entrepreneurship. There are branches in all nine provinces that assist people to start businesses or grow existing ones. Linked to these branches are enterprise information centres offering a range of useful information.

Tel.: 0860 103 703

Support for Cooperatives Programme
The Support for Cooperatives Programme promotes and develops cooperatives by providing training to both established cooperatives and people who are interested in starting one. Most cooperatives operate in agriculture, manufacturing, retail, construction, transport, textiles and clothing, and information and communications technologies.

Tel.: 012 394 1635/0861 843 384

Technology for Social Impact Programme
The Technology for Social Impact Programme focuses on the socio-economic development of rural communities, using indigenous knowledge and local resources to create economic opportunities.

Tel.: 012 317 4330

Development finance institutions (DFIs)

Industrial Development Corporation (IDC)
The IDC is a self-financing state-owned national DFI that provides financing to entrepreneurs and businesses engaged in competitive industries.

Tel.: 011 269 3000
Email: callcentre@idc.co.za
Website: www.idc.co.za

IDC Gro-E Youth Scheme
Tel.: 086 069 3888
Email: callcentre@idc.co.za
Website: www.idc.co.za/home/idc-products/special-schemes/gro-e-scheme/gro-e-youth-scheme.html

Development Bank of Southern Africa (DBSA)
The purpose of the DBSA is to accelerate sustainable socio-economic development by funding physical, social, and economic infrastructure. The bank plays multiple roles – financier, adviser, partner, implementer, and integrator – to mobilise finance and expertise for development projects.

Tel.: 011 313 3911
Email: webmaster@dbsa.org:
Website: www.dbsa.org

National Empowerment Fund (NEF)
The NEF promotes and facilitates black economic empowerment (BEE) and transformation. Its mandate and mission are to be a catalyst for broad-based BEE through asset management, fund management, and strategic projects.

Tel.: 011 305 8000
Email:info@nefcorp.co.za
Website: www.nefcorp.co.za

Small Enterprise Finance Agency (SEFA)
SEFA offers bridging loans, term loans, and structured finance through direct lending. Through its wholesale finance facility, SEFA provides finance to SMMEs through a network of micro finance institutions (MFIs) and retail finance institutions (RFIs) and offers credit guarantee products through partnerships with the banking sector.

Tel.: 086 000 7332
Email: helpline@sefa.org.za
Website: www.sefa.org.za

Independent Development Trust (IDT)
The IDT has a mandate to support government in meeting its social mandate to alleviate poverty by improving the quality of life of poor rural communities. It has earned a reputation for being a development programme implementing agency focusing on development planning, implementation, and coordination of government programmes.

Tel.: 012 845 2000
Email:info@idt.org.za
Website: www.idt.org.za

Micro Agricultural Financial Institutions of South Africa (MAFISA)
MAFISA was developed as a micro andretail agricultural financial scheme for economically active poor people. MAFISA allows access to financial services through selected institutions on an affordable and sustainable basis. It assists with loans to target groups, individuals, farmers, and other groups as well as savings and banking facilities at approved financial institutions.
Tel.: 012 319 7295

Other
BuddiBox
Entrepreneur development programme
Tel.: 087 150 7554
Website: www.buddibox.co.za

Itsoseng Women's Project
Waste Management Centre – Orange Farm
Tel.: 078 924 7139

Raymond Ackerman Academy
The Raymond Ackerman Academy is a post-matric-level academy that offers an inspiring and innovative six-month, full-time programme in entrepreneurial development.

Tel.: 011 559 5583
Website: www.ackermanacademy.co.za

Additional resource: Education and training

Universities

North-West University – Vaal Triangle Campus
Contact details for prospective students:
Vaal Triangle Campus

Tel.: 016 910 3111
Email: 10060073@nwu.ac.za
Website:
http://studies.nwu.ac.za/studies/apply

University of South Africa (Unisa)
Contact details for prospective students:

Call centre: 0861 670 411
Tel.: 011 670 9000
Email: study-info@unisa.ac.za
Website: www.unisa.ac.za

Universities of technology

Vaal University of Technology
Contact details for prospective students:
Tel.: 016 950 9000/0861 861888
Email: reception@vut.ac.za
Website: www.vut.ac.za

Technical and vocational education and training (TVET)

Sedibeng TVET College – Vereeniging
Tel.: 016 455 6500
Website: http://www.sedcol.co.za/

Sedibeng TVET College – Sebokeng
Tel.: 016 930 2800

Sedibeng TVET College – Vanderbijlpark
Tel.: 016 380 0300

Central Johannesburg TVET College
Tel.: 011 351 6000/ 6053
Email: info@cjc.edu.za
Website: www.cjc.edu.za

Sector education and training authorities (SETAs)

Sector education and training authorities (SETAs) are responsible for promoting skills development by increasing investment in education and training.

Agricultural Sector Education Training Authority SETA
Tel.: 012 301 5600
Email: info@agriseta.co.za
Website: www.agriseta.co.za

Bank SETA
Tel.: 011 805 9661
Website: www.bankseta.org.za

Construction Education and Training Authority SETA
Tel.: 011 265 5900
Website: www.ceta.org.za

Chemical Industries SETA
Tel.: 011 726 4026
Email: info@chieta.org.za
Website: www.chieta.org.za

Culture, Arts, Tourism, Hospitality, and Sports SETA
Tel.: 011 217 0600
Email: info@cathsseta.org.za
Website: www.cathsseta.org.za

Energy and Water SETA
Tel.: 011 689 5300
Email: info@eseta.org.za
Website: www.eseta.org.za

Education Training and Development Practices SETA
Tel.: 031 304-5930
Website: www.etdpseta.org.za

Financial and Accounting Services SETA
Tel.: 011 476 8570
Email: fassetcallcentre@fasset.org.za
Website: www.fasset.org.za

Fibre, Processing, and Manufacturing SETA
Tel.: 011 234 2311
Email: info@fpmseta.org.za
Website: www.fpmseta.org.za

Food and Beverage Manufacturing Industry SETA
Tel.: 011 253 7300
Email: info@foodbev.co.za
Website: www.foodbev.co.za

Health and Welfare SETA
Tel.: 011 607 6900
Email: hwseta@hwseta.org.za
Website: www.hwseta.org.za

Insurance SETA
Tel.: 011 544 2000
Email: insetacallcentre@inseta.org.za
Website: www.inseta.org.za

Local Government SETA
Tel.: 011 456 8579
Email: learnerships@lgseta.co.za
Website: www.lgseta.org.za

Media, Advertising, Information and Communication Technologies SETA
Tel.: 011 207 2600
Website: www.mict.org.za

Manufacturing, Engineering, and Related Services SETA
Tel.: 010 219 3000
Website: www.merseta.org.za

Mining Qualifications Authority SETA
Tel.: 011 630 3500
Email: learnerships@mqa.org.za
Website: www.mqa.org.za

Public Service SETA
Tel.: 012 423 5700
Email: abbeys@pseta.org.za
Website: www.pseta.org.za

Safety and Security SETA
Tel.: 086 110 2477
Email: callcentre@sasseta.org.za
Website: www.sasseta.org.za

Services SETA
Tel.: 011 276 9600
Email: customer@serviceseta.org.za
Website: www.serviceseta.org.za

Transport SETA
Tel.: 011 781 1280/0197
Email: webadministrator@teta.org.za
Website: www.teta.org.za

Wholesale and Retail SETA
Tel.: 012 430 4930
Email: wrseta@wrseta.org.za

Other

Massive OpenOnline Varsity (MOOV)
Digital skills training
Website: www.moov.life/orange-farm

MCD training
Technical and soft skills training
Tel.: 016 360 2388
Website: www.mcdtraining.co.za

Harambee Youth Employment Accelerator
Work-readiness programme
Website: http://harambee.co.za/

Additional resource: Financial assistance and incentive schemes

Government has prioritised the provision of financial assistance to deserving candidates.

National Student Financial Aid Scheme of South Africa (NSFAS)

NSFAS identifies eligible students and provides them with loans and bursaries to obtain higher and further education and training qualifications.

To access a bursary or loan from the NSFAS, you must apply at the HE institution where you intend to study.

Tel.: 0860 067 327
Email: info@nsfas.org.za
Website: www.nsfas.org.za

Funza Lushaka ursary Programme

The Funza Lushaka ursary Programme is a multi year programme that promotes teaching in public schools. Full cost bursaries are available to enable eligible students to complete a full teaching qualification in an area of national priority. ecipients of these bursaries will be required to teach at a public school for the same number of years that they received the bursary. Students who do not have access to the Internet must contact the Financial Aid Office at the institution where they have been accepted for teacher education studies for the necessary application forms and assistance.

Website: www.funzalushaka.doe.gov.za

Social work

Bursaries for those interested in studying social work are provided by the Department of Social Development.(The student applies for the bursary at the provincial department's offices.) These bursaries have a work-back condition; that is, the student works for the Department of Social Development for the number of years for which he/she received the bursary (or longer).

Contact person: Ms VuyelwaMngadi
Tel.: 012 312 7294
Email: VuyelwaM@dsd.gov.za

Contact person: Ms Kefiloe Bosman Tel.: 012 312 7223
Email: KefiloeB@dsd.gov.za

Incentive schemes

In an effort to increase industrial competitiveness and broaden the participation of enterprises in the economy, the Department of Trade and Industry (dti) provides financial support to qualifying companies in various sectors of the economy. Financial support is offered for various economic activities, including manufacturing, business competitiveness, export development, and market access, as well as foreign direct investment

SMME development incentives

Black Business Supplier Development Programme (BBSDP)

The BBSDP is a cost-sharing grant offered to small black-owned enterprises to assist them to improve their competitiveness and sustainability in order to become part of the mainstream economy and to create employment.

The BBSDP provides a grant to a maximum of R1 000 000 (R800 000 maximum for tools, machinery, and equipment and R200 000 maximum for eligible enterprises to improve their corporate governance, management, marketing, productivity, and use of modern technology).

Cooperative Incentive Scheme (CIS)

The CIS is a 90:10 matching cash grant for registered primary cooperatives. (A primary cooperative consists of five or more members who are historically disadvantaged individuals.) The CIS is an incentive for cooperative enterprises in the emerging economy to acquire competitive business development services. The maximum grant that can be offered to one cooperative entity is R350 000

Technology and Human Resources for Industry Programme (THRIP)

The THRIP is a partnership programme funded by the dti and managed by the National Research Foundation (NRF). On a cost-sharing basis with industry, THRIP supports science, engineering, and technology research collaborations focused on addressing the technology needs of participating firms and encouraging the development and mobility of research personnel and students among participating organisations.

Industrial development-related incentives

Business Process Services (BPS) Incentive
The South African government implemented a Business Process Outsourcing and Off shoring (BPO&O) Incentive Programme in July 2007. Between July 2007 and March 2010, the incentive programme resulted in the creation of at least 6 000 new jobs and attracted R303 million in direct investment. The BPS Incentive aims to attract investment and create employment in South Africa via off shoring activities.

Capital Projects Feasibility Programme (CPFP)
The CPFP is a cost-sharing programme that contributes to the cost of feasibility studies likely to lead to projects outside South Africa that will increase local exports and stimulate the market for South African capital goods and services.
The size of the grant must fall in the range of R100 000 to R5 million, to a maximum of 55% of the total cost of the feasibility study for projects in Africa and 50% for projects outside Africa.

Clothing and Textile Competitiveness Improvement Programme (CTCIP)
The CTCIP aims to build capacity among clothing and textile manufacturers and in other areas of the apparel value chain in South Africa to enable them to effectively supply their customers and compete on a global scale. Such competitiveness encompasses issues of cost, quality, flexibility, reliability, adaptability, and capability to innovate.

Manufacturing Investment Programme (MIP)
The MIP is a reimbursable cash grant for local and foreign-owned manufacturers who wish to establish a new production facility, expand an existing production facility, or upgrade an existing facility in the clothing and textiles sector.

Foreign Investment Grant (FIG)
The FIG compensates qualifying foreign investors for costs incurred in moving qualifying new machinery and equipment (vehicles excluded) from abroad to the Republic of South Africa.

Tourism Support Programme (TSP)
The TSP is a reimbursable cash grant that aims to support the development of tourism enterprises that will stimulate job creation and increase the geographic spread of tourism investment. The grant is for the establishment or expansion of tourism operations such as:

- accommodation services;
- passenger transport services;
- tour operators; and
- cultural, recreational, and entertainment services.

Production incentive (PI)

Under the PI, applicants can use the full benefit as either an upgrade grant facility or an interest subsidy facility, or a combination of both.
Eligible enterprises include:

- clothing manufacturers;
- textile manufacturers;
- cut, make, and trim operators;
- footwear manufacturers;
- leather goods manufacturers; and
- leather processors (specifically for leather goods and footwear industries).

Sector-Specific Assistance Scheme (SSAS)

The SSAS is a reimbursable 80:20 cost-sharing grant offering financial support to export councils, joint action groups, and industry associations. The maximum allocation per project is R1.5 million.

Support Programme for Industrial Innovation (SPII)

The SPII is a support programme of the dti, managed by the IDC. The SPII is designed to promote technology development in industry in South Africa through the provision of financial assistance for the development of innovative products and/or processes. The SPII focuses specifically on the development phase, which begins at the conclusion of basic research and ends when a pre-production prototype has been produced.

SEDA Technology Programme (STP)

As part of the government's strategy to consolidate small-enterprise support activities since April 2006, the activities of the Godisa Trust, the National Technology Transfer Centre (NTTC), the three business incubators of the dti, the Technology Advisory Centre (TAC), the technology-transfer activities of the Technology for Women in Business (TWIB) programme, and the support programmes for small enterprises of the South African Quality Institute were merged into a single programme: the Seda STP. Benefits include subsidising R800 000 for tools, machinery, and equipment on a 35:65 cost-sharing basis and R200 000 for business development services on a 50:50 cost-sharing basis.

Women Economic Empowerment Incentives Bavumile

South African women are gifted and talented in designing and crafting fashionable products for both the local and the international markets, mainly promoting South African culture and heritage. However, one of their greatest challenges is to produce quality products that can easily occupy the shelves of many local and international retail shops and boutiques, thus catching the eye of the buyer. Bavumile seeks to ensure the quality production

of commercially viable products that are produced by women by imparting relevant skills and expertise.

Isivande Women's Fund (IWF)

IWF is an exclusive women's fund established by the dti. The fund aims to accelerate women's economic empowerment by providing more affordable, usable, and responsive finance.

The IWF targets formally registered enterprises, where 60% is owned and/or managed by women.

The enterprises must have been in existence and operation for two or more years and must fall in a loan range of R30 000 to R2 million.

Trade, export, and investment incentives

Critical Infrastructure Programme (CIP)

The CIP is a cost-sharing cash grant for projects designed to improve critical infrastructure in South Africa. The grant covers qualifying development costs from a minimum of 10% to a maximum of 30% towards the total development costs of qualifying infrastructure. It is made available to approved eligible enterprises on completion of the infrastructure project concerned. A cash grant to a maximum of 30% capped at R30 million for the development cost for qualifying infrastructure is available.

Export Marketing and Investment Assistance (EMIA)

- National pavilions at international events

The dti assists South African exporters by organising national pavilions to showcase local products at international trade exhibitions.

- International trade exhibition assistance

The dti provides financial assistance to export councils, industry associations, provincial trade and investment-promotion agencies, joint action groups, export clubs, and chambers of commerce for international trade exhibitions where no national pavilion has been scheduled/ approved.

- Group outward-selling missions

The dti provides assistance to South African exporters who aim to conclude export orders with foreign buyers. These missions are organised by export councils, chambers of commerce, provincial investment promotion agencies, export clubs, or the dti.

Film and Television Incentive

The South African government offers a package of incentives to promote its film production industry. The incentives consist of the Foreign Film and Television Production Incentive to attract foreign-based film productions to shoot on location in South Africa and the South African Film and Television Production and Co-Production Incentive, which aims to assist local film producers in the production of local content.

Additional resource: Career services (CV, job placement services)

National Youth Development Agency (NYDA)

The National Youth Development Agency (NYDA) has been established primarily to tackle challenges with which the nation's youth are faced. The NYDA plays a leading role in ensuring that all major stakeholders, that is, government, the private sector, and civil society, prioritise youth development and contribute to identifying and implementing lasting solutions that address youth development challenges. The NYDA provides the following products and services:

- Career guidance
- JOBS Database and Matching Service
- Support for cooperatives
- Youth volunteering
- Business opportunities support services
- Mentoring

It also offers the following programmes:

- NYDA | IDC | sefa Youth Fund
- Solomon Mahlangu Scholarship Fund
- NYDA Grant Programme
- Youth Build Programme
- National Youth Service Programme
- Entrepreneurship Development Programme
- Volunteer Business Mentorship Programme
- Business Consultancy Service (Voucher) Programme

Tel.: 0800 52 52 52
Email: info@nyda.gov.za
Website: www.nyda.gov.za

NYDA Youth Advisory Centres
Johannesburg Full-Service Branch
Tel.: 011 834 7660
Physical address: 17 Diagonal Street, Old JSE Building, Ground Floor, Newtown, Johannesburg, 2001

Boipatong Local Youth Office
Tel.: 016 988 9487
Physical address: 1990 Lekoa Street, Boipatong, 1911

Bophelong Local Youth Office
Tel.: 016 986 3711
Physical address: 5050, Extension 10, Bophelong, Sebokeng, 1913

Vaal University Local Youth Office
Tel.: 016 950 9496
Physical address: Educity Campus, Sebokeng, 1983

Sebokeng Thusong Service Centre
Tel.: 016 988 1960
Physical address: Emfuleni Local Municipality, Sedibeng District, 88 Moshoeshoe Street, Sebokeng, 1983

Thusong Service CentresWebsite: www.thusong.gov.za

Bophelong Thusong Service Centre Tel.: 016 986 3711
Physical address: Emfuleni Local Municipality, Sedibeng District, 5050, Extension 10, Bophelong

Orange Farm Thusong Service Centre
Tel.: 011 935 6492
Physical address: Johannesburg Metropolitan Municipality, 15747, Orange Farm, Extension 4 (next to SAPS), Orange Farm, 1805

Career Development Advice and Guidance Services (DHET)

Tel.: 0860 35 66 35 / 086 999 0123
SMS 072 204 5056 with your name and contact number to have a career adviser call you back

Additional resource: Support institutions

Lifeline services are free of charge and are available in your community.

Tirisano Wellness Centre
Residents from Palm Springs and surrounding areas
Physical address:
Palm Springs Mall
Corner of R1558 and Falcon Road
Palm Springs
Tel.: 016 581 1711

Khensani Wellness Centre
Residents from Tshepiso, Sharpeville and surrounding areas
Physical address:
Govan Mbeki Centre 2013
Phase 2
Tshepiso
Tel.: 076 867 6593

Mamello Wellness Centre
Residents from Sebokeng
Physical address:
Lesabasaba Primary School
Zone 13
Sebokeng
Tel.: 016 594 7166

Lifeline Johannesburg
Tel.: 011 728 1347
WhatsApp counselling line: 065 989 9238

Lifeline National Counselling Line
Tel.: 0861 322 322 (24 hours, seven days a week)
Email: lifeline@lifelinejhb.org.za
Website: www.lifelinejhb.org.za

5.1 Ways to present myself

In order to develop your career, it is important that others know what you want and can do. This is often important to others (future employers, training coordinators, etc.) who have to accept and assess you. It is, therefore, important that you can show your passions, values, strengths, and skills to these people. How do you show these to others?
Below, write down the ways that are effective for you.

Passion:

....

The way I do this:

....

....

....

Skill 1:

....

The way I do this:

....

....

....

Skill 2:

....

The way I do this:

....

....

....

5.2 Tips for good listening, speaking, and writing

Tips for good listening

- See whether you understand what the speaker means (Summarise, or ask clarifying questions).
- Show that you want to listen and understand (eye contact, body language, or nods).
- Ask the speaker for an explanation, if necessary.
- Be patient; do not interrupt the speaker.
- Do not directly give your own opinions and thoughts, unless the speaker asks for these.
- ....

Tips for speaking well

- Focus on your main point (Be short and persuasive).
- Check whether the listener has understood you by pausing after a few sentences everytime.
- Talk slowly and comprehensibly.
- Give examples.
- Pay attention to good body language and eye contact.
- Choose your words carefully, and be respectful.
- ....

Tips for good writing

- Describe clearly why you are writing to someone (what you want from that person).
- Check carefully that the spelling is correct.
- End off appropriately.
- Do not use informal (spoken) language.
- Use paragraphs, full stops, and commas.
- ....

5.3 Tips for effective communication

Behaviour	Example	Select two behaviours that you are good at
Asking good questions	"What do you think about that?"<br>"How did you find out?"	
Clarifying and interpreting	"It sounds like you see yourself as someone who ..."<br>"If I listen to you, I feel that ..."	
Recognising ambiguity	"If I understand correctly, you would like to follow a computer course, but you seem afraid to register because you are scared that the course may be too difficult."	
Capturing non-verbal signals	You see that your girlfriend's mouth is trembling when she says she is OK. Out of empathy, you say "I can imagine how scared you must be".	
Knowing when to be quiet	You feel comfortable when your friend is telling you an emotional story. Even though she pauses for a while, your body language communicates that you are in no hurry.	
Rephrasing and summarising	Friend: "I'm very scared."<br>You: "You're feeling anxious now."	
Having a positive approach	Instead of attacking a person's behaviour, try to understand why he/she is behaving in that way. Accept him/her as he/she is.	
Knowing how and when to confront	You see that a friend is confused about a situation. You listen to everything he/she wants to say. Then you say, "I wonder whether what you're saying is not maybe contradictory...".	

5.4 Observing and giving feedback

Positive and clear feedback helps people to understand what they are good at, and it also encourages them to do it well again next time.

Positive things to pay attention to include the following:

- Keeping eye contact
- Communicating clearly
- Being friendly
- Having a nice smile
- Having a positive attitude
- Having a calm way of speaking
- Being relaxed
- Asking valuable questions
- Listening well
- Summarising well
- Indicating unspoken feelings

This shows the following, among other things:

- Confidence
- Concern
- Empathy and compassion
- Warmth
- Initiative
- Consideration
- Kindness
- Sincerity
- Courage

People learn new skills faster if attention is paid to what they do well instead of what they do not do well!

5.5 Roleplay

During this role play, we will practise effectively transferring a message and, thus, how to “sell” yourself. Divide into pairs. Decide who will play which role. One of you is the employer, and the other is you. After the first round, you will swap roles.

Employer
Embrace your role as well as possible: you are an employer, and the person opposite you must present himself/herself so well that you are convinced of his/her qualities. You may also ask critical questions.

You
Critically think of your good qualities and strengths: what are you really good at? These points are a good start for finding out what you can present to the employer in the role play. It is up to you to prove yourself and present yourself in an effective way! Beforehand, look at the tips in the previous exercises.

After both of you have had a chance to play the role of the employer and yourselves, you can discuss what you thought of each other's interview and what went well during the conversation. Also think about how certain messages were transferred effectively. Would you be able to do it again? How well could you “sell” yourself

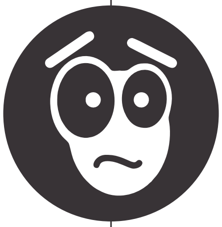

5.6 Facing my fears

What is holding you back?

....

....

Looking back on your career 20 or 30 years from now, what do you want to say you have accomplished?

....

....

What limiting beliefs do you hold about yourself and your ability to succeed inmaking your passion part of your life?

....

....

How have your fears and limiting beliefs held you back from finding or pursuing your passion in the past?

....

....

What solid evidence do you have that your fears and limiting beliefs are true?

....

....

Specifically, what actions can you take to lessen and manage your fear?

....

....

If there is some small amount of evidence that your fears or limiting beliefs might come true, is the risk big enough to prevent you from going after your passion?

....

....

What trumps finding and living your passion? Your current income? Your job? Your current lifestyle? Your home or the city in which you live? The opinion of others? How you spend your time? Something else?

....

....

Additional resource: The ultimate marketing tool: your CV

- **Personal details**
  Your name, surname, current telephone number, informal email address, and information regarding your driver's licence should be included here.
- **Career objective**
  An objective states your career focus and impresses the reader with your achievements and strengths.
- **Educational information**
  List brief details of your academic qualifications, starting with the most recent achievement/qualification. List the degree, diploma, matriculation, and the year, as well as the name of the institution.
- **Work-related experience**
  List your most recent experience first, continuing to your “oldest” experience. Mention the positions you have held and companies you have worked for, including the dates. Ensure that you include your part-time, volunteer, and vacation work. Mention the skills, abilities, and experience you have acquired during your work.
- **Skills**
  It is important to match your skills to the job for which you are applying. A CV that highlights skills and experience that match the job for which you are applying will boost your chances of getting an interview.
- **References**
  If this is your first job, it would be wise to nominate tutors or mentors as your referees. Always verify that they are willing to provide information about you before you include them in your CV.
- **Presentation and layout**
  The layout of the CV could already demonstrate whether you meet the skills specified in the job advertisement. The CV must be easy to read and must have a professional look. First impressions matter: use quality paper, and check for correct spelling and grammar.

Additional resource: Checklist for CVs

- Use high-quality paper, usually white, linen, light grey, or beige in colour.
- Your CV should be typed on a computer that produces clear, sharp type that will reproduce well.
- Your personal information: name, surname, email address, nationality, race, driver's licence (if you have one), and telephone number(s) are at the top of the page.
- Your career goal/objective: if you chose to use one, it appears directly under your personal information, so that the reader's eye is led to it immediately.
- Your career objective clearly states the functional areas in which you seek employment, the general level of the job you want, your eventual goals, and the specific skills set you would like to obtain.
- The objective should avoid using a job title (for example, personnel supervisor) when a more general designation (such as personnel management) would allow you to be considered for a wider variety of openings at several different levels.
- If you are applying for a specific job, use the exact job title.
- Organise the contents of the accomplishments section – if you choose to use it – to emphasise results produced, significant achievements, and recognition from others, whenever possible.
- List duties and responsibilities that show your specific skills and accomplishments.
- Include everything directly related to your specific job objective.
- Include education – formal and continuing – that supports your job objective.
- The CV should be interesting to read as well as pleasing to the eye. Your enthusiasm and energy should come across to the reader.
- Mention awards or community and social activities that support your job objective.
- Mention accomplishments that reflect your versatility or other exceptional qualities.
- Avoid writing negatively about previous employers.
- Avoid listing too many unimportant, meticulously detailed duties such as "opened mail, sorted correspondence".
- Exclude all extraneous data such as your religion, salary, weight, height, etc.
- Check the spelling of every word, and make sure that the grammar and punctuation are correct.
- All of the statements are presented in the same person and voice.
- All of the phrases begin with action verbs such as "supervised", "directed", "developed", "implemented", "organised", "planned", etc.
- The word "I" is used sparingly or not at all.
- Be truthful about your accomplishments rather than under emphasising them out of modesty or hyping them too much.
- Your CV must be visually attractive, interesting, clear, concise, easy to read quickly, and full of facts and details that prove that your experience and personal qualities would be assets to the organisation or the specific job for which you are applying.
- Your email address should be professional. Avoid nicknames.
- Do not change phone numbers.

Additional resource: Example of a CV

Curriculum Vitae Example
Kennith Khumalo

071 234 5678 · kennith.khumalo@gmail.com ·Postal address: PO Box 1234,Sebokeng, 1983

PERSONAL INFORMATION

Surname:	Khumalo
Name:	Kennith
IDnumber:	900101 0123 456
Nationality:	South African
Languages:	Tswana, Afrikaans, and English
Driver's licence:	Code B

SUMMARY

Write a short summary, and use words (adjectives) that will describe your personality, personal qualities, or traits that will lead to success in the specific job/career, as explained in the Qhubekela Phambili workshop.

- Enthusiastic, self-motivated, thoughtful teacher
- Specialising in creative curriculum planning and implementation
- Extensive management and organisational skills
- Sensitive and intuitive problem solver
- Proven flexibility in a variety of teaching situations
- Eagerness to learn

EMPLOYMENT HISTORY

Name of employer, period, job title, AND a description of what you did.

Sebokeng Primary School –Head of Science **(2013 – present)**

Duties entail:
- Performing the duties of a teacher and as the Head a Science;
- Ensuring good professional practice, standards, and quality of teaching through proper dialogue with the class teachers;
- Advising and contributing to curriculum development at school;
- Setting examination papers, co-ordinating marking schemes and moderating examinations and assessment; and
- Ensuring timely and adequate provision of textbooks, materials, and equipment required for the effective teaching.

Sebokeng Primary School –Science teacher **(2011 – 2013)**

Duties entailed:
- To ensure that all lessons are planned with clear aims and objectives;
- To ensure that all lessons are delivered in line with the departmental schemes of work and school policies;
- To encourage all students to be actively engaged in their own learning and in reviewing their progress against targets;
- To encourage and foster intellectual curiosity;
- To deal with inappropriate behaviour quickly and effectively according to the school behaviour policy;
- To set appropriate tasks and challenges for homework according to the published timetable, and ensure that it is regularly marked with constructive feedback; and
- To keep careful records of student progress in line with departmental and school policy.

Jack and Jill Learning Centre – kindergarten teacher **(2007 – 2010)**

Duties entailed:
- Design and follow a teaching plan;
- Teach alphabet and numeracy along with personal, social and emotional skills;
- Organise learning material and resources;
- Use a variety of activities and instructional methods (songs, stories, media, structured games, art, outdoor activities etc.) to motivate and stimulate children's abilities;
- Maintain an open line of communication with parents and provide appropriate information; and
- Follow and comply with teaching standards and safety regulations.

EDUCATION

- List qualifications, starting from highest, and include only the subjects that are necessary.
- List any courses attended or skills obtained.
- List any academic achievements.

Institution:	North-West University
Qualification obtained:	BEd Foundation Phase
Duration of studies:	2007 –2010

Institution:	Sebokeng High School, Sebokeng
Subjects passed:	Tswana<br>English<br>Mathematics<br>Physical Science<br>Accounting<br>Economics
Year of matriculation:	2006

Additional courses

Institution:	South African Institute of Science
Qualification obtained:	Certificate in Physical Science
Duration of studies:	2008

REFERENCES

Headmaster:	Mr P Johnson<br>Sebokeng Primary School<br>Sebokeng<br>082 222 3344
Lecturer:	Dr J Alison<br>Department of Education, NWU<br>Vanderbijlpark<br>082 234 5678

Additional CV tips

- The font should be easy to read, and the size should be between 10 and 12. Avoid colour.
- Your address could also be in table format below your name and surname.
- Your email address should be professional. Avoid nicknames. Highlight briefly how your skills and abilities fit the vacancy. Organise your experience in categories.
- Make sure that you include the institution, degree, date of completion, and main subjects. Start with the most recent qualification. It might not be necessary to include school subjects.
- The older you are, the less important your school information will become.

TIPS

Additional resource: Tips for making your list of job references

It is a good idea to prepare a list of references as part of your job search. Choose people who know you, who know about your work, and who would give you a good recommendation.

Make a list of people who know you and know about your work.
Past employers, supervisors from paid and volunteer positions, colleagues, and teachers.

Decide who you will ask to serve as a job reference.
Choose people who you think would give you a positive reference and who hold a position that others would respect. Refrain from using family members or friends as referees.

Contact these people by phone or in person.

- Start with a greeting, and give your name.
- Tell the person you are looking for a job.
- Let him/her know what type of job you are looking for.
- Ask whether he/she would be willing to be listed as a job reference.
- Ask whether he/she is aware of any job openings in your field.
- Send him/her a copy of your CV and any other helpful details.

Include the following for each person on your list:

- Full name/title
- Organisation and address
- Phone number
- How that person knows you

Type your list on good-quality paper. You will be prepared when an employer asks for your list of references. This list will also help you in filling out the reference section of an application.

Write "Thank you" notes to any referee who is called by a potential employer.

Periodically, you may want to let your referees know how your job search is going and ask whether they have heard of any job openings.

TIPS

Additional resource: Suggestions for writing a cover letter

A cover letter can be used to introduce your CV, and it provides an opportunity to highlight how your specific skills match the job requirements. It can also be used in a "letter writing" campaign when you are looking for possible job openings.

Present a good appearance.
The cover letter should look professional, printed on the same good-quality paper that you used for your CV.

Make it perfect.
Proofread your letter with care. Make sure that it is free of typing, spelling, or grammatical errors. You may want to ask someone else to review your letter and check for errors.

Use a business correspondence format.
Any business correspondence format will be fine. Centre the letter attractively on the paper, and make it no longer than one page.

Write to a specific person
Make sure that you have the correct spelling of the person's name, his/her title, and the correct address. Call and verify this information if you are not sure.

Make the content brief and focused.
Personalise your cover letter for each person you write to. Most people are not impressed by a standard letter.

1. Use an attention-grabbing first sentence. As the name suggests, an attention-grabbing sentence is designed to grab the reader's attention. This sentence should indicate that you know something about the company and how you could benefit it.
2. Say what attracted you to this particular company.
3. Include a paragraph that highlights your experience, skills, and accomplishments that are relevant to the job and that shows how they will benefit the employer. To do this, you need to know something about the position or the company to which you are writing. Analyse the advertisement to identify the requirements.
4. Include a few sentences about your training and personal traits that make you valuable to the potential employer.
5. State your next step. Do not leave it up to the employer to contact you.
6. Thank the person for his/her time and consideration.

TIPS

Additional resource: Example of a cover letter

Curriculum Vitae Example
Kennith Khumalo
071 234 5678 · kennith.khumalo@gmail.com ·Postal address: PO Box 1234, Sebokeng, 1983

22 April 2018

Damien Dice
Headmaster
Orange Farm Primary
222 Flamingo Road
Orange Farm
1841

Dear Mr Dice

Hope this letter finds you in good health. I am writing this letter to apply for an available vacancy, as advertised on Careers24. I believe my qualifications meet the requirements mentioned in the job profile. I attach my CV for your consideration.

I qualified as a primary school teacher ata respected university in 2010. I have worked as a science teacher at a local school, and I have always been respected by my colleagues and students. I am an enthusiastic teacher and love what I do. I also continuously attend training to keep up to date with trends and to constantly improve my teaching skills.

I hope to hear from you. Thank you in advance for your consideration.

Yours sincerely

Kennith Khumalo

Additional resource: Informational interviewing

What is it?
It is a form of networking in which the job seeker visits various work sites and talks to people employed in jobs that he/she is interested in learning more about.

How can it help?
It helps people interested in a career change learn about different jobs.
It is a source of possible job leads.
It helps job seekers prepare for job interviews.

What should I ask?
The following are questions you might consider asking when you interview people for information:

- How did you get into this line of work?
- What do you like most about your job?
- What do you like least about your job?
- Where else can I find people who do this type of work?
- What is it like to do this job? Is there a daily routine?
- What personal characteristics are the best fits for this job?
- What types of skills and abilities does this job require?
- What qualifications are needed for this kind of work: education, training, experience, licence/credentials, union memberships?
- Is there ongoing training? Can you describe it?
- How would you describe the supervision given to a person in this job?
- What equipment or business machines do you use?
- What is the pay range for a job in this field?
- Would it be possible to tour the worksite (if appropriate)?
- What do you predict will be happening in this field in the next five to 10 years?
- What are the opportunities for promotion?
- How would a person with my background find a job in this field?
- What other people or organisations do you recommend that I contact for more information about jobs in this field? May I mention your name when I make these contacts?

Additional resource: Tips for conducting an informational interview

Before

- Prepare questions before you go.
- Take a notebook to take notes.
- Get good directions.
- Arrive five to 10 minutes early.
- Dress neatly and appropriately.
- Avoid heavy perfumes and aftershaves; many people are allergic to them.
- Take a few copies of your CV; they may come in handy.
- Relax, this is not a job interview. It will be fun.

During

- Smile and greet the receptionist.
- Give your name and the name of the person you are there to see.
- Avoid smoking, chewing gum, or looking at papers on anyone's desk.
- When you meet the person you will be interviewing:
    - smile;
    - introduce yourself; and
    - offer a handshake.
- Make some upbeat small talk, perhaps about the weather.
- Express enthusiasm about being there, and begin the interview.
- Ask the questions you have prepared.
- Take notes on the important points.
- Stick to the agreed time (usually about 20 to 30 minutes).
- Ask the person who else he/she would recommend you talk to in that field.
- Ask whether you could use his/her name as a referral.
- Comment on something specific you have learnt as a result of the interview.
- Thank the person for his/her time.

After

- Keep track of your informational interviews in a notebook.
- Send a written "Thank you" note to the person you interviewed.
- Follow up on leads and referrals you received at your interview.
- Acknowledge and appreciate yourself for taking the initiative to conduct an informational interview.

TIPS

Additional resource: Pre-interview checklist

[ ] Research the company as much as possible
[ ] Try to know about the company, the job, the interviewer, and the salary range for the type of work in which you are interested.
[ ] Prepare a response to "Tell me about yourself".
[ ] Prepare answers to commonly asked interview questions and difficult questions.
[ ] Prepare concrete examples to support the skills that match the job requirements.
[ ] Practise being interviewed with friends, family, a mirror, etc.
[ ] Wear clean clothes that are appropriate for an interview.
[ ] Take along a notebook, pen, and other materials, copies of your CV, work samples, and your list of references.
[ ] Know the name of the person conducting the interview.
[ ] Get good directions.
[ ] Know how long it will take to get there.
[ ] Plan to arrive on time.
[ ] Plan to arrive at the site 20 minutes before the interview to allow for traffic and other delays. Arrive at the exact location of the interview five minutes before the interview is to begin.
[ ] Give yourself a pep talk.
[ ] Imagine a successful interview!
[ ] Relax, and breathe.

TIPS

Additional resource: Post-interview checklist

Company: ______________________________

Name and title of interviewer: ______________________________

Position interviewed for: ______________________________

Date of interview: ______________________________

Check each item you remembered to do during the interview.
Put a star (*) next to those items that you want to work on improving for the next interview.

[ ] Arrived on time.
[ ] Dressed neatly and appropriately.
[ ] Didn't chew gum or smoke.
[ ] Friendly to everyone at the workplace.
[ ] Greeted interviewer by name:
- Firm handshake
- Eye contact
- Good posture

[ ] Positive attitude.
[ ] Described my skills using concrete examples.
[ ] Closed interview by:
- summarising how my skills could benefit the employer;
- asking whether the employer had concerns about hiring me;
- determining next steps; and
- thanking the inter viewer and expressing enthusiasm about working there.

Additional resource: Follow-up plan

[ ] Write the next steps and the date to follow up in my plan book.

[ ] Send the interviewer an upbeat "Thank you" letter, thanking him/her for the interview and restating my interest in the job.

[ ] List what I did well during the interview and would like to repeat:

....

....

....

[ ] Questions I thought I answered well:

....

....

....

[ ] Questions I would like to answer better:

....

....

....

[ ] Other areas I would like to improve:

....

....

....

Additional resource: Dressing for the interview

First impressions are important.
The initial impression you make on an interviewer can affect the rest of the interview. You can make a positive impression by doing the following:

- A firm handshake
- A warm smile
- Relaxed body language
- Dressing well!

Dress in a way that suits you.
Wear colours in which you look good and feel comfortable. Wear patterns and colours that coordinate. Ask people whose fashion sense you respect to give feedback on your appearance.

Dress in a way that is appropriate for the job.
Consider the image you want to present and what type of clothing is suitable for the job. For instance, a suit and tie would probably not be appropriate when interviewing for a position as a plumber.

Dress in a way that respects the interviewer.
Consider the interviewer, and dress in a way that shows he/she is important.

Some general guidelines:

- Wear clothes that fit well and are clean and well ironed.
- A neat, attractive, conservative hairstyle.
- Clean well-groomed fingernails. Avoid glittery nail polish and bright colours.
- Briefcases, purses, pens, notebooks, or anything else that you take to the interview should be in good shape and present a professional image.
- Jewellery should be simple and conservative. For men, unless you know the interviewer will not be offended, it is best to leave your earring at home.
- Avoid heavy perfumes and aftershaves. Scent is personal and may offend the interviewer or even trigger an allergic reaction.
- If you are bothered by bad breath, take a breath mint or brush your teeth before entering the interview.
- Try to visit a bathroom with a mirror before your interview to check your appearance.

Additional resource: Organising your job search

The following suggestions can help you turn what you know into productive action.

Establish a work area for yourself.
At the very least, this area should include:

- an ample amount of table space and a place to sit;
- space to store your job-search materials; and
- a telephone.

Helpful job-search materials:

- Pens
- Business-size paper
- Business-size envelopes
- Postage stamps
- Phone books/address book
- Calendar/plan book
- Updated copies of your CV
- Updated copies of applicable credentials
- Letters of recommendation
- Names and numbers of referees
- Licences, work samples
- Access to a computer
- "Thank you" cards

See page 54 and 55 for career counselling services that offer these services free of charge or a ta minimal rate.

Schedule your time.
It can be easy to get side-tracked by other activities. It is important to establish a job-search schedule and stick to it!

Pace yourself, and set realistic goals!

1. Decide how many hours per week you will spend on your job search.
2. Decide which days and which hours you will look for work.
3. Decide how many calls you will make per day.
4. Use a calendar or other planning tool to keep track of the hours you spend on your job search.

A daily job-search schedule can help you stick to your plan. Schedule your time much the same way as you would a job.

A sample schedule:

07:30-08:30	Wake up, shower, dress, eat breakfast
08:30-09:00	Plan your job-search strategies
09:00-10:30	Implement strategies
10:30-10:45	Take a break
10:45-12:30	Implement strategies
12:30-13:30	Lunch
13:30-15:30	Continue implementing

Plan your job-search strategies.
Getting job leads
- Contact people in your personal job-lead network.
- Ask family and friends whether they are aware of any job openings in your field.
- Newspapers:
  - Read want advertisements (Apply even if you do not meet all the requirements).
  - Read local business sections for new companies or expansions in your area.
- When you are interviewed and are not offered the position, ask the interviewer for job leads.
- Find job hotline numbers, and call weekly, as jobs are updated.
  - Most universities, hospitals, government agencies, and schools and many private industries have job hotlines. Look in the phone book or in books in bookstores/libraries.
- Use a computer online service to find job openings and as a source of networking.
  - Many public libraries, non-profit career counselling services, and state agencies offer free online access.
- Prepare a brief summary of your skills. Include the type of work you are looking for, your name, and your phone number. Type the information on 3x5 cards. Carry these with you, and hand them out to people you meet. They can also be given as reminders to people in your personal job-lead network.
- Conduct informational interviews.
- Read trade magazines.

More job-search strategies:
- Prepare for interview questions.
- Practise answering interview questions.
- Call and request interviews.
- Make job-lead phone calls.
- Track phone calls.
- Mail follow-up letters, CV's, and "Thank you" notes.

Enlist assistance from others.
Family: your immediate family also probably experiences the stress of your job search. Asking them for assistance can help them feel more involved and less helpless. It can also make your job search easier.
- Ask for assistance with household tasks.
- Ask for cooperation with transportation.
- Ask for assistance with typing and copying.
- Ask them to proofread materials.

- Tell them what to say when they answer the phone and how to take messages.
- Ask members of your household to maintain quiet when you get job-related calls.
- Ask for the emotional support you need.
- Try to be understanding of the stress your family experiences.
- Remember to thank family members for their support and assistance.
- Practise being interviewed with family members.

Friends: you many want to enlist a “job support buddy” who:
- calls you at an agreed-on time each week;
- talks to you for 15 to 30 minutes;
- asks how your job search is going and how much time you have spent on your job search; and
- offers support and encouragement.

It is remarkable how talking to someone about your job-search activities regularly can help you stay on track. (This person could also be a family member, but it is best if this person is not emotionally involved in your getting a job).

Commit yourself to finding a job. Your job search is your temporary full-time job!

Additional resource: Social media for professionals

The world as we know it is changing day by day. One of the not so recent changes is the way we use social media. Most people check their social media platforms when they get up, in their lunch hour, in between and when they go to bed.

In our free time we upload pictures, tell people where we are by checking in, tweeting our opinions and showing people what we are having for dinner. It's as if we have the constant need to stay connected and share the information about ourselves into the virtual world. This is a great way to communicate, share memories and staying connected. But when you upload something, have you ever thought about what the implications it can have on your job or even when you start applying for a job? Most people forget that they are publishing content into a public domain where most of the time your content is available for the world to see.

We sometimes forget that prospective employers are also on these social media platforms and that they are using these platforms to search for an employee and to eliminate employees before hiring.

So basically you should consider what you post. Be careful!

Use the "mother rule" before posting. This rule basically says that do not post any content that you are not willing to share with your mother. If you have a very open relationship with your mother you should use a different reference. Stay away from racial remarks, drunken pictures, sexual content, hate speech or defamation of something or someone.

But do not refrain from using social media. An excellent social media tool to use as an online CV platform is LinkedIn. LinkedIn is basically Facebook for professionals. What makes LinkedIn cool is you can add all your professional experience to one place. This allows people from outside to view your profile and connect with you if they want to. On LinkedIn you can search for jobs and connect with employers who might be hiring.

A LinkedIn CV is always available and can be printed out when you need it. This means that you always have a CV online and can update it as you go along.

Additional resource: Tips for completing forms / documents adequately

Here are tips for completing any type of document adequately.

- Ensure that you plan to complete, review, and return your document before the deadline. It is a common error to rush completion the night before a deadline.
- Read and follow the instructions carefully. Always take a few minutes to review the entire document before starting.
- Carefully read all supporting documents before you start.
- When completing documents electronically, keep the font size and spacing consistent. If you are not able to complete the form electronically, make sure that you complete it as neatly as possible. In both instances, make sure that the tense is consistent.
- If there are questions that do not apply to you, simply respond with “not applicable” or “n/a”. Don't leave any blanks.
- Consider whether a question is closed, asking for specific information (for example, “Yes” or “No”), or whether it is open, where a clear, succinct response is expected.
- Proofread your document before sending/submitting it. Check for any spelling or grammar errors that may not be picked up by an automatic spellchecker.
- Keep copies of your documents for future reference.

6.1 I commit myself to…

6.3 Final statement

What steps will I take for my career in the coming week/month?

What am I going to do?

-..

-..

-..

What steps will I take for my career in the next six months? What am I going to do?

-..

-..

-..

Do I really follow the agreements I have made with myself?

Then I will reward myself with the following:

-..

-..

-..

Date: ....

Signature: ....

TIPS

My name: ______________________________

6.4 What I have learnt from…/What I admire about…

Finish the sentence. Think of what you have learnt from, or like about, your fellow participants.

TIPS

6.5 What I want to let go

A thousand-step journey begins with a single step!

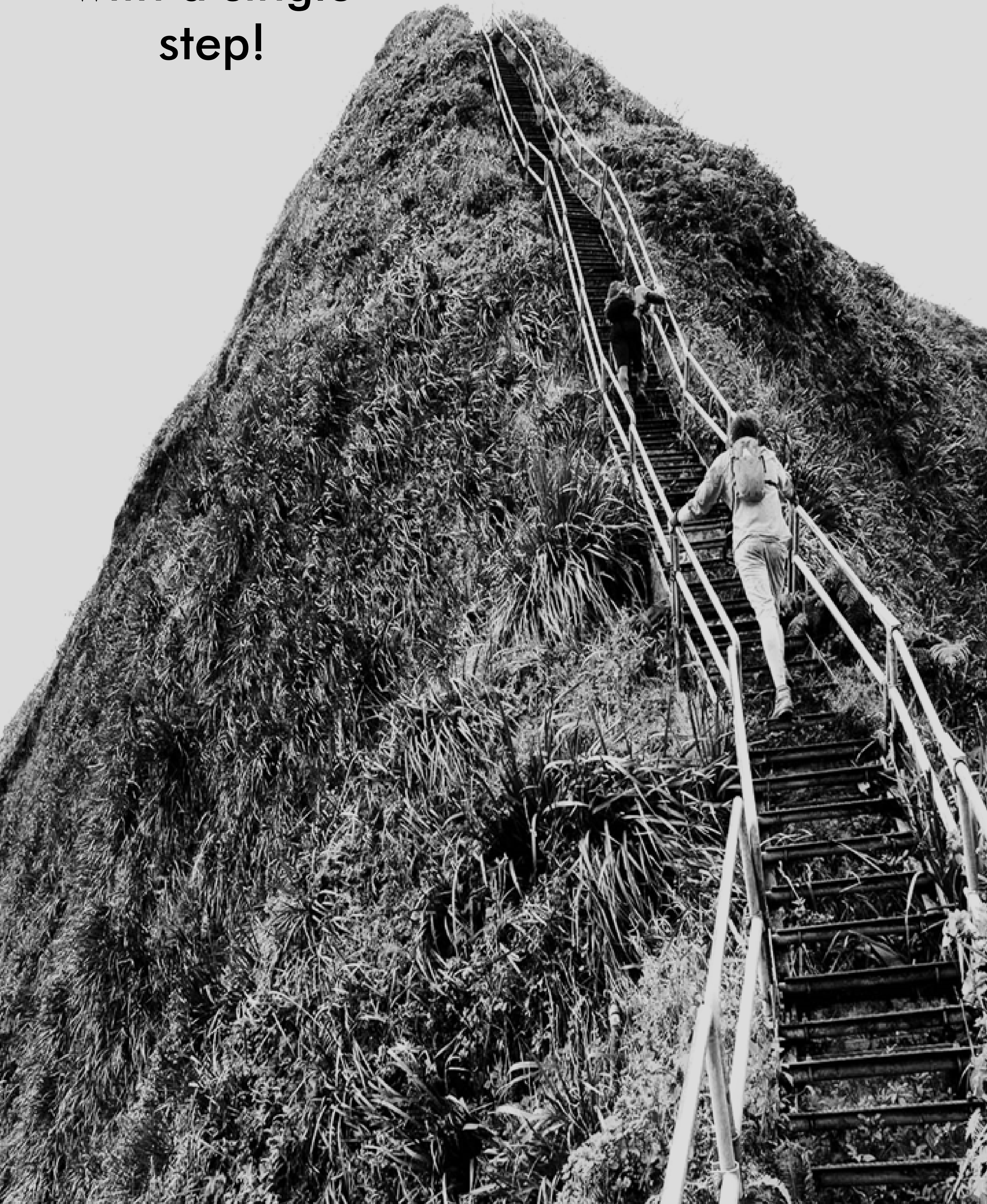

Some final thoughts

Some of the most sought-after skills, competencies, and traits I wish someone had told me about earlier in my job:

- Your career is yours and yours alone. Don't expect any handouts.
- Make sure that you have a map and that you know where you want go.
- Use the resources that are available.
- Make sure that you have the right knowledge, skills, and attitudes in your toolbox.
- Be excited about your journey, and love what you do; if you don't, no one else will.
- If you get stuck on your journey, don't give up and lose hope. No one ever reached the top without setbacks.
- Get experience; use any opportunity to learn.
- Remember that your destination is always changing. You never stop learning and growing. As soon as you reach a goal, set a new exciting goal.
- Be willing to grow in many ways other than just in your job over the next few years. Keep an open mind!
- Don't be afraid to make mistakes, as they don't define who you are. Work is so much more than achievements.
- Good luck with your journey. May it be filled with great success.

Qhubekela Phambili Team

References

Academic Development and Support. (2016). *Career Centre Career Guide*. North-West University.

Akkermans, J. & Lagerveld, S. (2010). CareerSKILLS: *Protocol van een interventie gericht op het versterken van loopbaancompetenties van jonge werknemers*. [CareerSKILLS: Protocol of an intervention aimed at strengthening career competencies of young employees]. Netherlands: TNO.

Curran, J., Wishart, P. & Gingrich, J. (1999). *JOBS: A manual for teaching people successful job-search strategies.* Michigan Prevention Research Center, Institute for Social Research, University of Michigan, Ann Arbor, MI.
